# Influenza vaccination reveals and partly reverses sex dimorphic immune imprints associated with prior mild COVID-19

**DOI:** 10.1101/2022.02.17.22271138

**Published:** 2022-02-22

**Authors:** Rachel Sparks, William W. Lau, Can Liu, Kyu Lee Han, Kiera L. Vrindten, Guangping Sun, Milann Cox, Sarah F. Andrews, Neha Bansal, Laura E. Failla, Jody Manischewitz, Gabrielle Grubbs, Lisa R. King, Galina Koroleva, Stephanie Leimenstoll, LaQuita Snow, OP11 Clinical Staff, Jinguo Chen, Juanjie Tang, Amrita Mukherjee, Brian A. Sellers, Richard Apps, Adrian B. McDermott, Andrew J. Martins, Evan M. Bloch, Hana Golding, Surender Khurana, John S. Tsang

**Affiliations:** 1 Multiscale Systems Biology Section, Laboratory of Immune System Biology, NIAID, NIH, Bethesda, MD, USA; 2 Graduate Program in Biological Sciences, University of Maryland, College Park, MD, USA; 3 NIH Center for Human Immunology, NIAID, NIH, Bethesda, MD, USA; 4 Division of Intramural Research, NIAID, NIH, Bethesda, MD, USA; 5 Vaccine Research Center, NIAID, NIH, Bethesda, MD, USA; 6 Division of Viral Products, Center for Biologics Evaluation and Research (CBER), FDA, Silver Spring, MD, USA; 7 Laboratory of Clinical Immunology and Microbiology, NIAID, NIH, Bethesda, MD, USA; 8 NIH Clinical Center, NIH, Bethesda, MD, USA; 9 Department of Pathology, Johns Hopkins University School of Medicine, Baltimore, MD, USA; 10 These authors contributed equally

## Abstract

Viral infections can have profound and durable functional impacts on the immune system. There is an urgent need to characterize the long-term immune effects of SARS-CoV-2 infection given the persistence of symptoms in some individuals and the continued threat of novel variants including the recent rapid acceleration in infections. As the majority of COVID-19 patients experienced mild disease, here we use systems immunology approaches to comparatively assess the post-infection immune status (mean: 151 [5^th^ – 95^th^ percentile: 58 – 235] days after diagnosis) and subsequent innate and adaptive responses to seasonal influenza vaccination (as an “immune challenge”) in 33 previously healthy individuals after recovery from mild, non-hospitalized COVID-19, as compared to 40 age- and sex-matched healthy controls with no history of COVID-19. Sex-specific, temporally stable shifts in signatures of metabolism, T-cell activation, and innate immune/inflammatory processes suggest that mild COVID-19 can establish new post-infection immunological set-points. COVID-19-recovered males had an increase in CD71^hi^ B-cells (including influenza-specific subsets) before vaccination and more robust innate, influenza-specific plasmablast, and antibody responses after vaccination compared to healthy males. Intriguingly, by day 1 post-vaccination in COVID-19-recovered subjects, the expression of numerous innate defense/immune receptor genes (e.g., Toll-like receptors) in monocytes increased and moved away from their post-COVID-19 repressed state toward the pre-vaccination baseline of healthy controls, and these changes tended to persist to day 28 in females, hinting that the acute inflammatory responses induced by vaccination could partly reset the immune states established by prior mild COVID-19. Our study reveals sex-dimorphic immune imprints and *in vivo* functional impacts of mild COVID-19 in humans, suggesting that prior COVID-19 could change future responses to vaccination and in turn, vaccines could help reset the immune system after COVID-19, both in an antigen-agnostic manner.

## Introduction

Infection with SARS-CoV-2 can result in persistent clinical sequelae for months after initial infection, both in those requiring hospitalization and those with mild disease^1^. While the spectrum of clinical manifestations associated with post-acute COVID-19 syndrome (a.k.a “long COVID”) is expanding, understanding the molecular and cellular immunological changes associated with recovery from SARS-CoV-2 infection is lacking, particularly in those with less severe, non-hospitalized disease, the population that constitutes the majority of COVID-19 recoverees. Important questions include how “homeostatic”/baseline immune states may have been altered by the infection, and whether any alterations may affect responses to future challenges (such as infection or vaccination). Examples of long-term immunological effects of viral infection have previously been described, e.g., following natural measles infection there is marked reduction in humoral immunity and increased susceptibility to various non-measles infections for months to years^2,3^. A better understanding of whether even mild SARS-CoV-2 infection could result in persistent immunological changes that may affect future immune responses has important public health implications given the large number of infected individuals in the world (more than 370 million global cases as of February 2022; covid19.who.int)^4,5^. Thus, we enrolled and comparatively analyzed using systems immunology approaches healthy, non-obese individuals who: 1) recovered from non-hospitalized, mild cases of COVID-19, and 2) age- and sex-matched controls who never had COVID-19. In addition to assessing the post-COVID-19 immunological states, we utilized seasonal influenza vaccination to evaluate the immune responses of these two populations at the serological, transcriptional, proteomic, and cellular levels.

## Results

**Figure 1.**
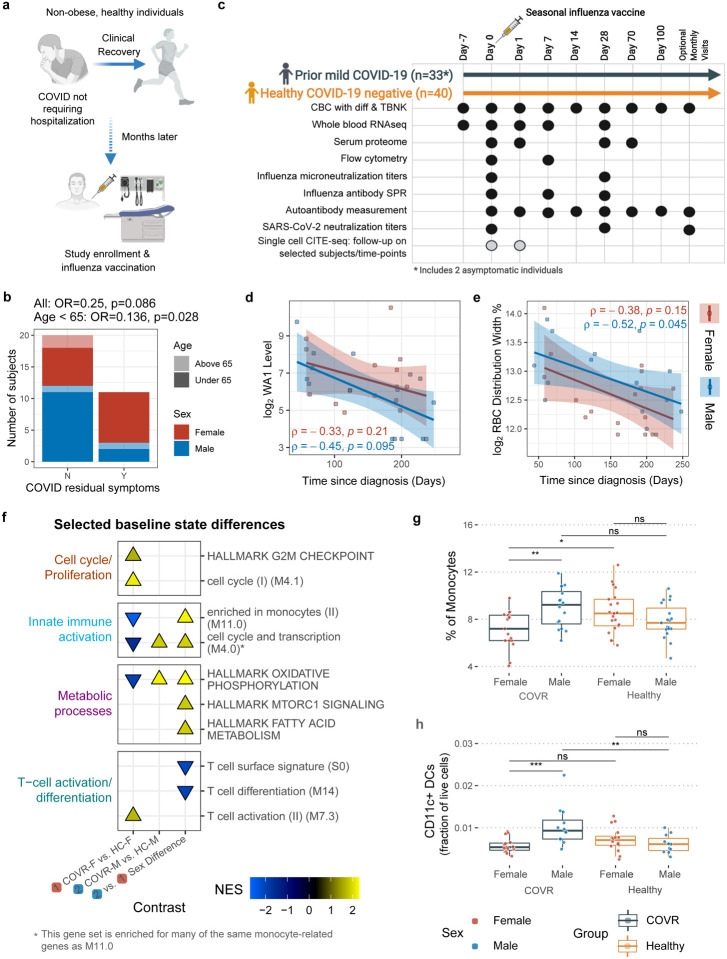
Study overview and evaluation of baseline (before influenza vaccination) molecular and cellular differences in COVID-19-recovered subjects. **a**, Schematic showing the study design. **b**, Bar plot showing the proportion of COVID-19-recovered (COVR) subjects with residual symptoms (see Extended Data Table 1) at the time of study enrollment following recovery from non-hospitalized COVID-19. OR = odds ratio of the likelihood of having residual symptoms between female (F) and male (M). P-value determined by two-tailed Fisher’s exact test. **c**, Data generated at each timepoint in the study. CBC with diff & TBNK = Complete Blood Count with Differential and T- and B-Lymphocyte and Natural Killer Cell Profile; SPR = Surface plasmon resonance **d**, Scatterplot showing the correlation between the time since diagnosis in days (TSD; x-axis) and the SARS-CoV-2 neutralization titer for COVR subjects (y-axis) at day 0 (D0) prior to influenza vaccination. Spearman’s rank correlation and p values are shown. **e**, Similar to (**d**), but showing the correlation between the TSD (x-axis) and the red blood cell distribution width (RDW) at D0 (y-axis). **f**, Blood transcriptomic analysis of the stable baseline (before influenza vaccination) differences among COVR and healthy control (HC) groups. Enrichment plot showing the normalized enrichment scores (GSEA NES) of selected gene sets of the different comparisons (GSEA FDR < 0.05; see Methods; see Extended Data Table 4 for all significant gene sets with FDR < 0.05). The NES are plotted separately for COVID-19-recovered females (COVR-F) versus healthy control females (HC-F), COVID-19-reocovered males (COVR-M) versus healthy control males (HC-M), or the difference between the two sets of comparisons (COVR-M versus COVR-F taking healthy sex differences into account). Positive NES (upward arrow) indicates that gene set scores are higher in the first group than the second group listed in the comparison; negative NES (downward arrow) indicates that gene set scores are higher in the second group than the first group listed in the comparison. Only gene sets not correlated with time since diagnosis across COVR subjects at baseline are considered as stable. **g**, Box plots comparing the percentage of monocytes in peripheral blood (y-axis) between COVR-F (n = 17), COVR-M (n = 16), HC-F (n = 21), and HC-M (n = 19) at baseline (average of day −7 and D0). Significance of differences is determined by two-tailed Wilcoxon test. * p ≤ 0.05 and ** p ≤ 0.01; ns = not significant **h**, Similar to (**g**) but for the proportion of CD11c+ dendritic cells (as the fraction of live cells; y-axis) between COVR-F (n = 15), HC-F (n = 16), COVR-M (n = 12), and HC-M (n = 11) at D0. Significance of differences is determined by two-tailed Wilcoxon test. ** p ≤ 0.01 and *** p ≤ 0.001; ns = not significant.

Individuals with prior symptomatic SARS-CoV-2 infection (diagnosed by nasal PCR test) or asymptomatic infection (by antibody test; see Methods), and age- and sex-matched healthy controls with no history of COVID-19 were recruited from the community (Fig. 1a, see Methods). For those with a history of COVID-19 the average time since diagnosis was 151 days (5^th^ – 95^th^ percentile: 58 – 235 days after diagnosis; Extended Data Table 1), including two individuals who had asymptomatic COVID-19 infection, defined as positive for antibodies against SARS-CoV-2 with no history of symptoms or positive nasal PCR test (and not enrolled in COVID-19 vaccine trials). All COVID-19-recovered individuals had clinically mild illness during acute disease that did not require hospitalization (self-reported average length of illness: 20.8 days), nor did they have any major medical comorbidities, to include infection at the time of enrollment, obesity (BMI > 30) or autoimmune disease. A small number of individuals continued to have mild sequelae from their illness at study enrollment (3 males and 8 females), the most common being loss of taste and/or smell (Fig. 1b, Extended Data Table 1). Females tended to be more likely to have sequelae (p = 0.09 for all subjects, p = 0.03 for those < 65 years of age), at a rate similar to that reported in other larger studies^6^.

### Prior mild COVID-19 is associated with stable sex-specific molecular and cellular differences

**Extended Data Figure 1.**
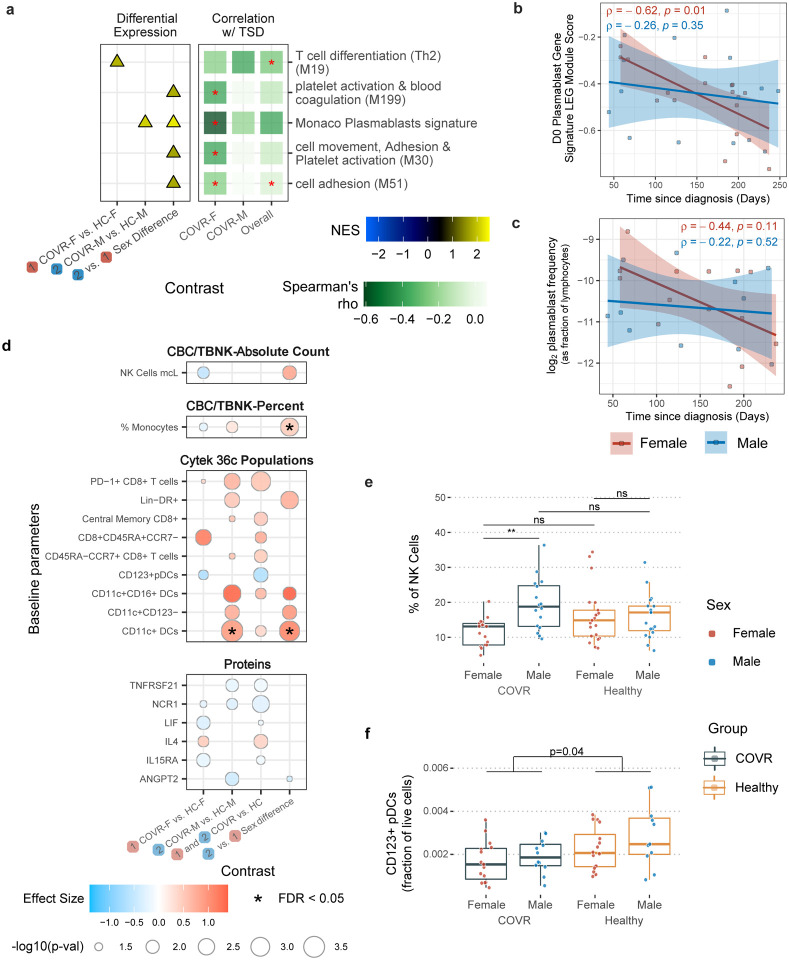
Baseline (pre-vaccination) molecular and cellular differences between COVID-19-recovered subjects and healthy controls. **a**, Blood transcriptomic analysis of the baseline (before influenza vaccination) differences between COVID-19-recovered (COVR) and healthy control (HC) groups that are associated with the time since diagnosis (TSD) in COVR subjects (see Methods). (left) Enrichment plot showing the baseline normalized enrichment scores (GSEA NES) of selected gene sets of the different comparisons (GSEA FDR < 0.05; see Methods; see Extended Data Table 4 for all significant gene sets with FDR < 0.05). The NES are plotted separately for COVR females (COVR-F) versus HC females (HC-F), COVR males (COVR-M) versus HC males (HC-M), or the difference between the two sets of comparisons (COVR-M versus COVR-F taking healthy sex differences into account). Positive NES (upward arrow) indicates that gene set scores are higher in the first group than the second group listed in the comparison; negative NES (downward arrow) indicates that gene set scores are higher in the second group than the first group listed in the comparison. (right) Spearman’s correlation of gene set module scores [using leading edge genes (LEGs)] with the TSD separately in COVR-F, COVR-M, and both groups combined. * p < 0.05 in linear models accounting for age and race (see Extended Data Table 2). **b**, Scatterplot showing the correlation between the TSD (x-axis) and the plasmablast gene signature LEG module score (see Methods; y-axis) at day 0 (D0). Spearman’s rank correlation and p values are shown. **c**, Similar to (**b**), but for the plasmablast (CD27+CD38+) frequency from flow cytometry. **d**, Bubble plot showing significant (p < 0.05) cell frequency and circulating protein baseline differences of the indicated comparisons. The complete blood count (CBC) and lymphocyte phenotyping (TBNK) are shown in the top two boxes [including day −7 (D-7) and D0], followed by D0 Cytek spectral 36-color flow cytometry panel (middle box), and D0 OLINK proteomic platform (bottom box). Only those with an unadjusted p-value of < 0.01 in at least one of the comparisons are shown (see Methods; Extended Data Table 3 for full results). * FDR < 0.05 (adjusted within each panel and comparison). **e**, Box plots comparing the percentage of natural killer (NK) cells in peripheral blood as measured in lymphocyte phenotyping panel (TBNK; y-axis) between COVR-F (n = 17), COVR-M (n = 16), HC-F (n = 21), and HC-M (n = 19) at baseline (average of D-7 and D0). Significance of group difference is determined by two-tailed Wilcoxon test. ** p ≤ 0.01; ns = not significant **f**, Similar to **(e)** but comparing the proportion of pDCs (as percentage of live cells; y-axis) between COVR-F (n = 15), HC-F (n = 16), COVR-M (n = 12), and HC-M (n = 11) at D0. Significance of COVR vs. HC difference is determined by two-tailed Wilcoxon test.

Multi-omics profiling was performed using whole blood transcriptomics, serum protein profiling, antibody characterization, and peripheral blood immune cell frequencies with hematological parameters from a complete blood count (CBC) and clinical and research flow cytometry (Fig. 1c, Supplementary Information Fig. 1, Supplementary Information Table 1; TBNK: CD4+ and CD8+ T-cells, B-cells, NK cells). Consistent with previous reports, SARS-CoV-2 neutralizing antibody titers negatively correlated with time since COVID-19 diagnosis (TSD) in both COVID-19-recovered males (COVR-M) and females (COVR-F) (Fig. 1d)^7^. Because immunological resolution following infection may unfold over time after symptoms subsided, we first asked which parameters continued to evolve following mild SARS-CoV-2 infection. We focused on parameters that differed between COVID-19-recovered individuals and healthy controls (HCs) but were correlated with TSD (Extended Data Tables 2 and 3, see Methods). Among the hematological parameters, the red cell distribution width (RDW), a measure of the variation of erythrocyte volume, was negatively correlated with TSD in COVR-M and trended similarly in COVR-F (Fig. 1e). As elevated RDW is observed in cases of hematological dysregulation^8,9^, this suggests that even mild COVID-19 may disrupt hematopoiesis, resolution of which may take weeks to months, consistent with reports of persistently altered erythrocyte deformability 4–8 months after hospitalization for SARS-CoV-2 infection^10^. Several other TSD correlates among all COVID-19-recovered subjects were sex-specific (Extended Data Fig. 1a), consistent with earlier findings that the acute immune responses to COVID-19 are sex dependent^11–19^. For example, plasmablast-related gene transcription and frequency were negatively correlated with TSD, decreasing over time in females but not apparent in males (Extended Data Fig. 1b, c); thus, plasmablast transcriptional signatures were on average elevated in COVR-M compared to COVR-F (Extended Data Fig. 1a). Since antibody titers against SARS-CoV-2 were declining in both sexes, these circulating plasmablast-like cells probably no longer made or secreted antibodies. Similarly, there was higher expression of platelet activation and cell adhesion genes in COVR-M compared to COVR-F (after taking sex differences in healthy individuals into account; Extended Data Fig. 1a), partly because expression of these genes declined over time in the COVR-F but not in COVR-M, suggesting that these platelet and cell adhesion related gene expression changes following SARS-CoV-2 infection were progressively resolving in COVR-F but might represent an unresolved, temporally stable “immune state” in COVR-M (at least over the time scale of our examination).

**Extended Data Figure 2.**
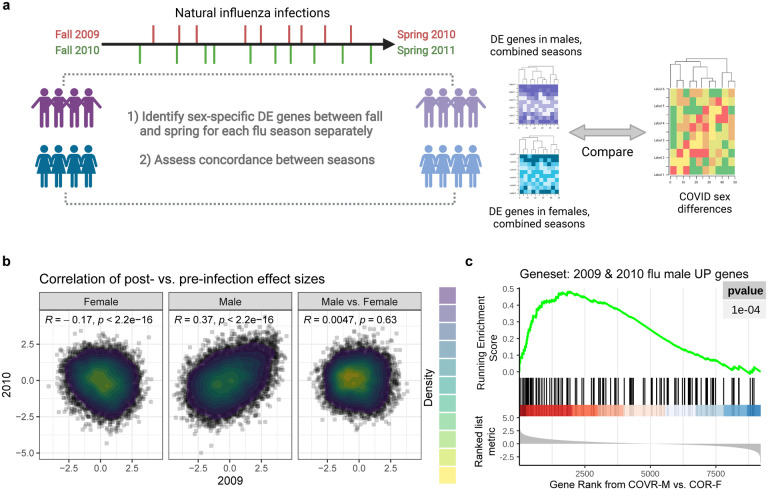
Persistent post-infection gene expression changes following natural influenza infection. **a**, Schematic showing the approach used to evaluate changes in blood gene expression before and after natural influenza infection published in Zhai *et al* (2015), and how those gene changes may relate to sexspecific differences resulted from prior COVID-19 in this study. **b**, Density plot showing the correlation between the gene expression changes (see Extended Data Table 5) before (fall) and after (spring) natural influenza A infection in 2009 (x-axis) and 2010 (y-axis) for females (left), males (center), and male vs female contrast (right). Shown are Spearman’s rank correlation and p values. **c**, Gene set enrichment plot of the genes that are upregulated in men between fall (pre-infection) and spring (post-infection) in both 2009 – 2010 and 2010 – 2011 seasons. Genes were ranked by the signed log10(p-value) in the COVID-19-recovered male vs COVID-19-recovered female contrast at baseline using only subjects under 65 years of age. The tick marks denote the location of the genes in the influenza gene set.

To further examine sex-dependent immune states associated with prior mild COVID-19, we systematically evaluated differences in COVID-19-recovered males and females compared to their respective matched healthy controls that are not significantly associated with TSD: 1) COVR-F vs. HC females (HC-F); 2) COVR-M vs. HC males (HC-M); and 3) sex differences: differences between COVR-M and COVR-F after accounting for male-female differences in HCs (Fig. 1f, Extended Data Fig. 1d, Extended Data Tables 3 and 4). The sex differences include depressed T-cell-related but elevated innate immune cell activation transcriptional signatures (Fig. 1f), and increased frequencies of monocytes, conventional/myeloid dendritic cells (cDCs), and NK cells (Fig. 1g, h, Extended Data Fig. 1e). The frequencies of monocytes and cDCs in COVR-M were elevated to levels similar to those of healthy females and, in the case of cDCs, significantly higher than COVR-F and healthy males (Fig. 1g, h). There were also sex differences in metabolic transcriptional signatures, including oxidative phosphorylation and the mechanistic target of rapamycin (mTOR) complex 1 (mTORC1) signaling (Fig. 1f). Together, these data suggest that immune recovery from mild COVID-19 differed between males and females, with COVR-M exhibiting temporally stable elevations in myeloid cell frequencies, and innate immune activation and metabolic transcriptional signatures, while COVR-F had higher transcriptional signatures of T-cell differentiation/activation and cell cycle, but lower monocyte frequency than their male and healthy counterparts. Supporting our finding that natural respiratory viral infections may lead to unresolved sex-specific “immune states”, we found persistent changes following community influenza infection in males but not females by using a published blood transcriptomic dataset on pre- and post-natural influenza A [predominantly pandemic H1N1 (pH1N1)] infection in individuals followed longitudinally over the course of two influenza seasons^20^ (Extended Data Fig. 2a–c, Extended Data Table 5); the genes with increased expression in males were also enriched for genes more highly expressed in COVR-M compared to COVR-F in our cohort (after accounting for the expected sex differences present in healthy subjects), although in general such imprints are likely pathogen dependent. This observation provides additional support that exposure to a respiratory viral pathogen can potentially lead to persistent immunological imprints detectable in blood, even in healthy individuals with mild disease. In contrast to the numerous sex-specific differences observed in our data, we detected far fewer sex-independent differences (i.e., comparing COVID-19-recovered vs. healthy alone with both sexes combined) in our cohort. Among the few were depression of plasmacytoid dendritic cells (pDCs) in both COVR-M and COVR-F (Extended Data Fig. 1f), which is consistent with a previous report^21^ and perhaps a remnant of the apoptotic/stress state and lower frequencies of peripheral pDCs found during acute COVID-19^22,23^. Together, our findings suggest that even mild, non-hospitalized SARS-CoV-2 infections may establish new, temporally stable, sex-dependent immunological imprints.

### Prior mild COVID-19 is associated with both innate and adaptive responses to influenza vaccination

We next asked whether these post-COVID-19 immune state differences may affect an individual’s ability to respond to future, non-SARS-CoV-2 immunological challenges. The seasonal influenza quadrivalent vaccine was administered to study participants, who were subsequently followed longitudinally for up to 100 days to evaluate the immune response to the vaccine at the serological, molecular, and cellular levels (Fig. 1a, 2a). The cellular and molecular responses to seasonal influenza vaccination have been well characterized in healthy adults, including transcriptional and cellular changes in blood that reflect the activation and interaction of distinct cell populations and pathways in innate and adaptive immune cells. These include early innate/inflammatory and interferon (IFN) responses on day 1 after vaccination and a strong but transient plasmablast peak around day 7 culminating in the generation of influenza-specific antibodies and memory cells^24–27^. Thus, influenza vaccination provides an excellent model of coordinated immune activity to probe the functional impacts of prior mild SARS-CoV-2 infections.

**Extended Data Figure 3.**
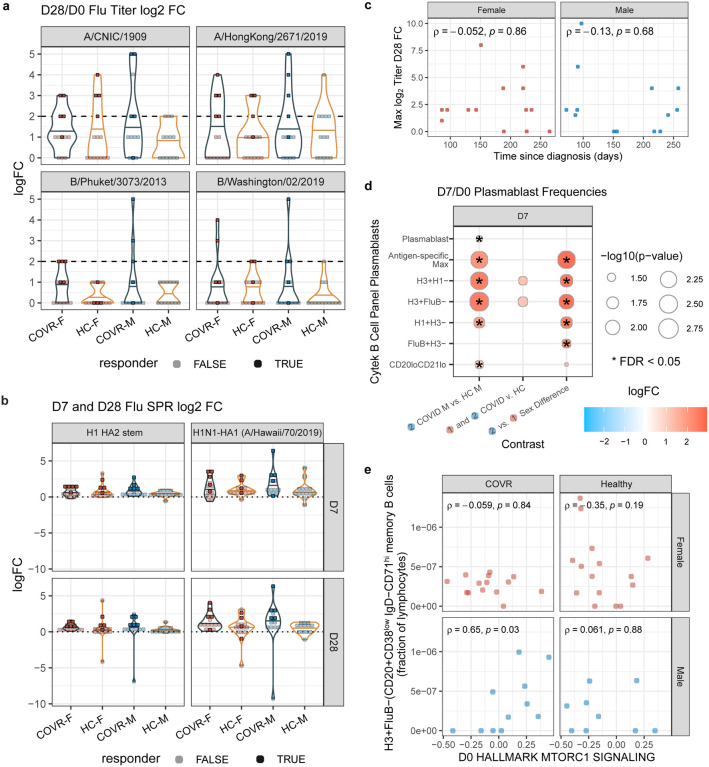
Serological and B-cell responses to the influenza vaccine. **a**, Violin plot of the [day 28 (D28)/day 0 (D0)] influenza strain-specific log fold change (FC) for each of the four strains in the seasonal influenza vaccine. Each column shows the response for a separate group of subjects, under 65 years of age, in the study: blue dots = males (M), red dots = females (F). Gray outline = COVID-19-recovered (COVR) subjects. Orange outlines = healthy control (HC) subjects. Dark circle indicates a strong responder to the vaccine, defined as responding to 2 or more of the 4 vaccine strains with a (D28/D0) fold change of 4 or greater. **b**, Similar to (**a**), but showing the log fold change for [day 7 (D7)/D0] (top) and [D28/D0] (bottom) surface plasmon resonance (SPR) measurements for the H1 HA2 stem (left) or H1N1-HA1 (A/Hawaii/70/2019). **c**, Scatterplot showing the correlation between the time since diagnosis in days (TSD; x-axis) and the (D28/D0) log fold change of influenza antibody titer (maximum of all four strains in the vaccine shown; y-axis). Spearman’s rank correlation and p values are shown. **d**, Bubble plot showing differential levels of the plasmablast frequencies at D7. A population is included if the difference is significant (p < 0.05) in at least one of the comparisons shown (see Methods). **e**, Scatterplots showing the correlation between the D0 frequency of H3+ FluB− CD71^hi^ memory (CD38^low^CD71^hi^IgD−) B-cells (as fraction of lymphocytes; y-axis) and D0 Hallmark MTORC1 Signaling gene set module score (x-axis) for COVR females (COVR-F; top left, n = 14), HC females (HC-F; top right, n = 15), COVR males (COVR-M; bottom left; n = 11), and healthy control males (HC-M; bottom right, n = 9). Spearman’s rank correlation and p values are shown.

**Figure 2.**
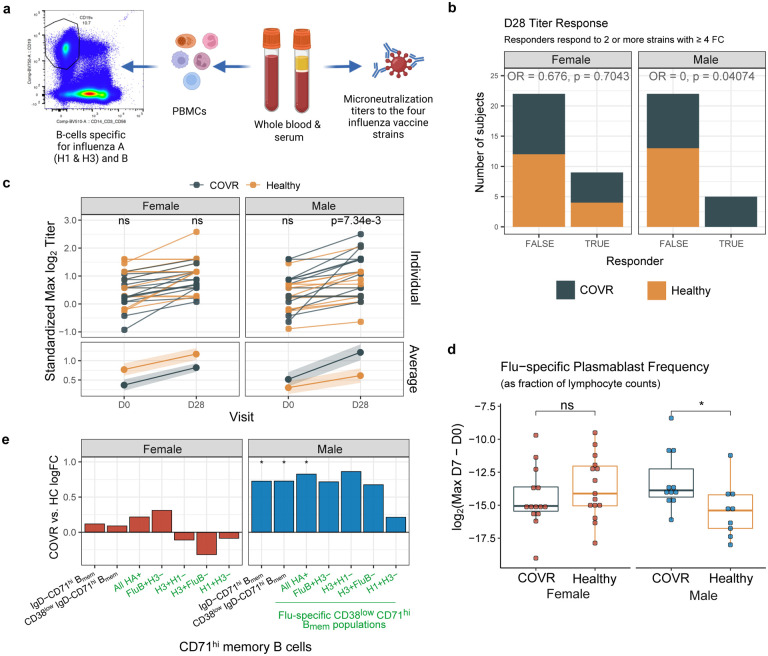
Serological and B-cell responses to the influenza vaccine. **a**, Schematic of data generation. **b**, Bar plots showing the number of subjects, under 65 years of age, who responded to two or more influenza strains in the quadrivalent seasonal influenza vaccine with a fold change (FC) of 4 or more from day 0 (D0) value at day 28 (D28) after influenza vaccination (“responder”,see Extended Data Fig. 3a). Results are shown separately for COVID-19-recovered females (COVR-F) and healthy control females (HC-F) (left) and COVID-19-recovered males (COVR-M) and healthy control males (HC-M) (right). OR = odds ratio of sex-specific association between being a responder and COVID status. P-value determined by two-tailed Fisher’s exact test. **c**, Maximum normalized influenza vaccine titer (among the four strains in the vaccine) at D0 (prior to vaccination) and D28 after vaccination, shown separately for COVR-F and HC-F (left) and COVR-M and HC-M (right) under 65 years of age. The top shows the D0 and D28 values for individual subjects in the study. The bottom shows the average value for each group at the two timepoints. Shaded area represents standard error. Statistical significance of COVID-recovered (COVR) vs. healthy control (HC) difference at each timepoint was determined by linear regression models accounting for age, race, and influenza vaccination history (see Extended Data Table 6). ns=not significant (p > 0.05). **d**, Box plots comparing day 7 (D7) and D0 (baseline) difference of influenza-specific plasmablast (PB; CD27+CD38+CD20^low^CD21^low^) frequency as percentage of lymphocytes (maximum value among B+ H3−, H1+ H3−, H3+ B−, and H3+ H1− PBs), plotted separately for COVR-F (n = 14), HC-F (n = 15), COVR-M (n = 11), and HC-M (n = 9). Significance of group difference is determined by two-tailed Wilcoxon test. * p ≤ 0.05; ns = not significant. **e**, Bar plots showing the fold change in D0 (prior to influenza vaccination) frequency of CD71^hi^ memory (CD71^hi^IgD−) B-cells (as fraction of lymphocytes) as well as influenza-specific cells (with green label) between COVR and HC subjects, separately for females (left) and males (right). Significance was determined by linear regression models accounting for age, race, and vaccination history (see Extended Data Table 3). * p ≤ 0.05.

Among subjects ages 18–64, COVID-19-recovered individuals with persistent symptoms were more likely to experience vaccine adverse events (AEs; p = 0.02), including pain at the injection site and myalgia (no serious AEs were reported). Serological responses to vaccination were broadly intact in COVID-19-recovered subjects, with robust titer responses at Day 28, but sex-specific differences were again observed. COVR-M were more likely to be “high” responders compared to healthy males (Fig. 2b, c, Extended Data Fig. 3a), defined as responding to 2 or more of the 4 vaccine strains with a day 28/day 0 fold-change of 4 or greater [“seroconversion”^28^]. There was no relationship between prior COVID-19 infection and day 7 or 28 influenza antibody avidity (as measured by surface plasmon resonance^29,30^; Extended Data Fig. 3b) or between the TSD and day 28 titer responses in either males or females (Extended Data Fig. 3c).

Consistent with their more robust antibody responses, COVR-M had a higher increase of influenza-specific plasmablasts (as evaluated by the maximum change) than healthy males at day 7 (Fig. 2d, Extended Data Fig. 3d, Supplementary Information Fig. 2a). Intriguingly, we detected higher proportions of CD71^hi^ memory B-cells (CD38^low^CD71^hi^CD19+CD20+IgD−), including the influenza-specific memory B-cells contained within this subpopulation, at baseline (prior to vaccination) in COVR-M compared to HC-M (Fig. 2e, Supplementary Information Fig. 2b). A population of CD71^hi^ B-cells (termed “activated B-cells”) has been noted to emerge early after both vaccination and natural viral infection, can originate from the memory or naïve B-cell pools, and persists longer than antibody-secreting cells after a natural viral infection^31^. Our finding of elevated frequencies of CD71^hi^ B-cells prior to vaccination can perhaps be attributed to broad, antigen non-specific B-cell activation (including the influenza-specific subsets and likely other specificities) by prior SARS-CoV-2 infection; similar phenomena have been reported in vaccination and natural viral infection with measles and varicella^32,33^. Curiously, the proportion of these H3+ pre-vaccination CD71^hi^ memory B-cells was correlated with expression of several metabolic genes from the mTORC1 signaling pathway^34^ in COVR-M only (Extended Data Fig. 3e). This suggests that the increases in these B-cells could reflect a more activated metabolic state in COVR-M prior to vaccination. Together, these observations reveal that mild infection with SARS-CoV-2 can result in sex-specific phenotypic and functional immunological changes detectable months after disease, as exemplified by serological and influenza-specific B cell response alterations following immunization with non-SARS-CoV-2 antigens.

**Extended Data Figure 4.**
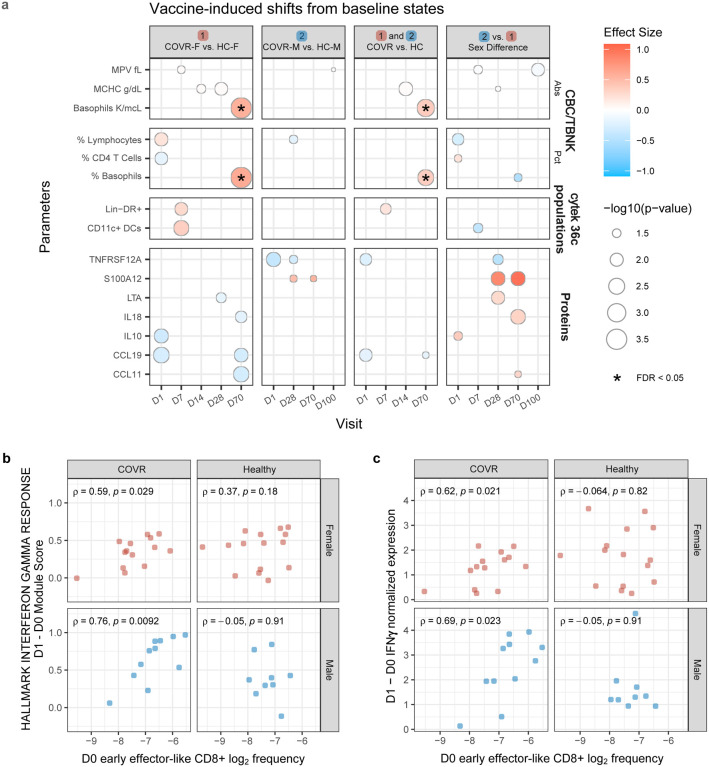
Changes in molecular and cellular parameters after influenza vaccination. **a**, Bubble plot showing vaccine-induced response in different comparison groups (shown on the top) at the days post vaccination (x-axis) relative to the pre-vaccination baseline. The parameters include complete blood count (CBC) and lymphocyte phenotyping (TBNK) parameters [top two rows; relative to day −7 and day 0 (D0)]; cell populations from the Cytek 36-color flow cytometry panel (middle row; relative to D0), and proteins from the OLINK platform (bottom row; relative to D0). A parameter is shown if the difference is significant at unadjusted p-value of < 0.01 in at least one of the comparisons shown at the top (see Methods; see Extended Data Table 6 for full results). D1 = day 1, D7 = day 7, D14 = day 14, D28 = day 28, D70 = day 70, D100 = day 100. **b**, Scatterplots showing the correlation between the D0 log_2_ frequency of early effector-like CD8+ T-cells (as fraction of live lymphocytes; x-axis) and (D1 - D0) Hallmark Interferon Gamma Response gene set module score (y-axis) for COVID-19-recovered females (COVR-F; top left, n = 14), healthy control females (HC-F; top right, n = 15), COVID-19-recovered males (COVR-M; bottom left; n = 11), and healthy control males (HC-M; bottom right, n = 9). Spearman’s rank correlation and p values are shown. **c**, Similar to (**b**) but showing the (D1-D0) serum IFNγ protein level from the OLINK platform (y-axis).

**Figure 3.**
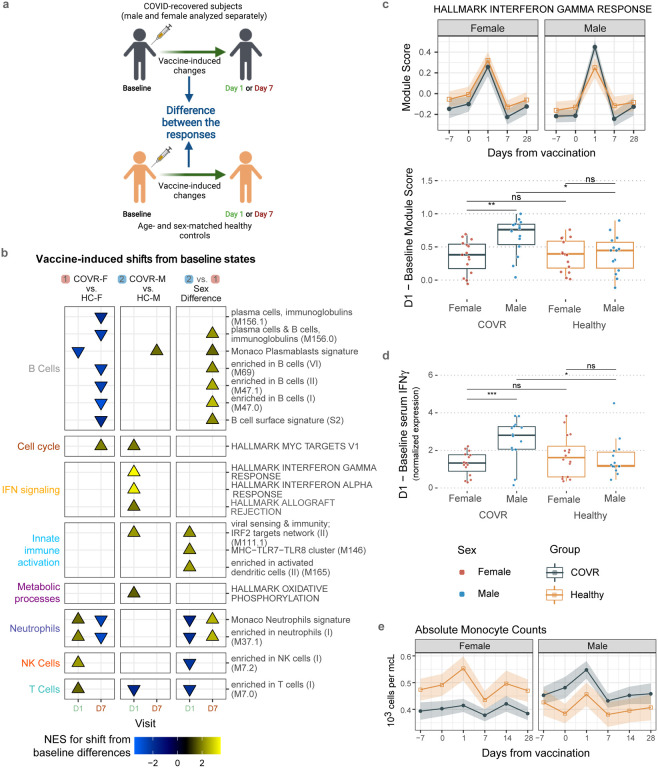
Sex-specific molecular and cellular response differences to influenza vaccination in COVID-19recovered individuals and matching controls. **a**, Schematic showing the sex-specific comparisons of influenza-vaccine induced changes from baseline (pre-vaccination) at early timepoints post vaccination [day 1 (D1) or day 7 (D7)] between COVID-19-recovered (COVR) subjects (in grey at top) and healthy control (HC) subjects (orange at bottom). These comparisons (difference of the within-group vaccine-induced differences) for blood transcriptomic data are plotted in (**b**). **b**, Similar to Fig. 1e but here showing the GSEA analysis comparing the early (D1 and D7) influenza vaccination responses in COVR vs. HC subjects for females (1), males (2), and sex differences [2 vs. 1; i.e., COVR males (COVR-M) versus COVR females (COVR-F) taking healthy sex differences into account] (see Methods). Plotted are the gene sets that show significant changes from the baseline [day-7 (D-7) and day 0 (D0)] within each comparison group [e.g., COVR-F and healthy control females (HC-F) for 1] and significant differences between the two groups at the indicated timepoints (FDR < 0.05; see Extended Data Table 7). Positive NES (upward arrow) indicates that gene set scores are higher in the first group than the second group listed in the comparison; negative NES (downward arrow) indicates that gene set scores are lower in the first group than the second group listed in the comparison. **c**, (top) Average module scores of the “Hallmark Interferon Gamma Response” gene set at various timepoints before (D-7 and D0) and after influenza vaccination (D1, D7 and day 28) separately for COVR (grey line) and HC (orange line) males (left) and females (right). The module scores were generated from the full gene set (gene count = 187). Shaded areas indicate standard error. (bottom) Box plots showing the (D1 – baseline) difference in the Hallmark Interferon Gamma Response module score for the subjects shown on the top, including COVR-F (n=15), COVR-M (n=14), and HC-F (n=16), and HC-M (n=14). Average of D-7 and D0 samples was used to represent baseline for each subject. Significance of group difference is determined by two-tailed Wilcoxon test. * p ≤ 0.05 and ** p ≤ 0.01; ns – not significant **d**, Box plots of the D1 response (D1 – D0) of serum IFNγ protein level from the OLINK platform for COVR-F (n=15), COVR-M (n=14), HC-F (n=16), and HC-M (n=14). Significance of group difference is determined by two-tailed Wilcoxon test. * p ≤ 0.05 and *** p ≤ 0.001; ns – not significant **e**, Changes in mean absolute monocyte count across timepoints for COVR (grey line) and HC (orange line) subjects separately for females (left) and males (right). Shaded areas indicate standard error.

We next assessed the day 1 and 7 blood transcriptomic, circulating protein, and cell frequency responses relative to baseline (days −7 and 0 prior to vaccination), separately in males and females (Fig. 3a, Extended Data Fig. 4a, Extended Data Table 6). At day 1 after vaccination, COVR-M had significantly stronger IFN transcriptional responses compared to both COVR-F and HC-M (Fig. 3b, c). Consistent with this, circulating IFNγ levels in serum were elevated in COVR-M on day 1 (Fig. 3d). The day 1 increase in the IFN gene signature and IFNγ protein level was more strongly correlated with the pre-vaccination (day 0) frequency of early effector-like CD8+ T-cells in COVR-M compared to COVR-F (Extended Data Fig. 4b, c), suggesting that some of the IFNγ might have emerged from this population potentially in response, in an antigen-agnostic manner, to the inflammation induced by the influenza vaccine^35^. Furthermore, in agreement with the higher level of influenza-specific plasmablasts observed at day 7 in the COVR-M (Fig. 2d), the plasmablast transcriptional signature was also elevated in this group compared to COVR-F, and conversely, several B-cell related gene sets had lower expression in COVR-F than males on day 7 (Fig. 3b, Extended Data Table 7). In contrast to males, COVR-F displayed stronger NK cell and neutrophil transcriptional response signatures on day 1 (Fig. 3b), but without apparent increases in monocyte frequencies like in males (COVR-M or HC-M) or healthy females (Fig. 3e) as typically observed in healthy influenza vaccinees [e.g., see^24,27^]. Even though myeloid cell frequencies, including monocytes, were already elevated in COVR-M before vaccination at baseline (Fig. 1f, 1g, 3e), a robust increase in monocytes on day 1 that subsequently reverted to baseline levels by day 7 and onwards was evident in the COVR-M (Fig. 3e). Thus, consistent with the sex-specific immune set points associated with prior mild COVID-19, the early innate and adaptive responses to the influenza vaccine, which is antigenically distinct from SARS-CoV-2, were also markedly different between COVID-19-recovered and healthy controls in a sex-dependent manner.

### Partial “reset” of gene expression imprints following influenza vaccination

**Extended Data Figure 5.**
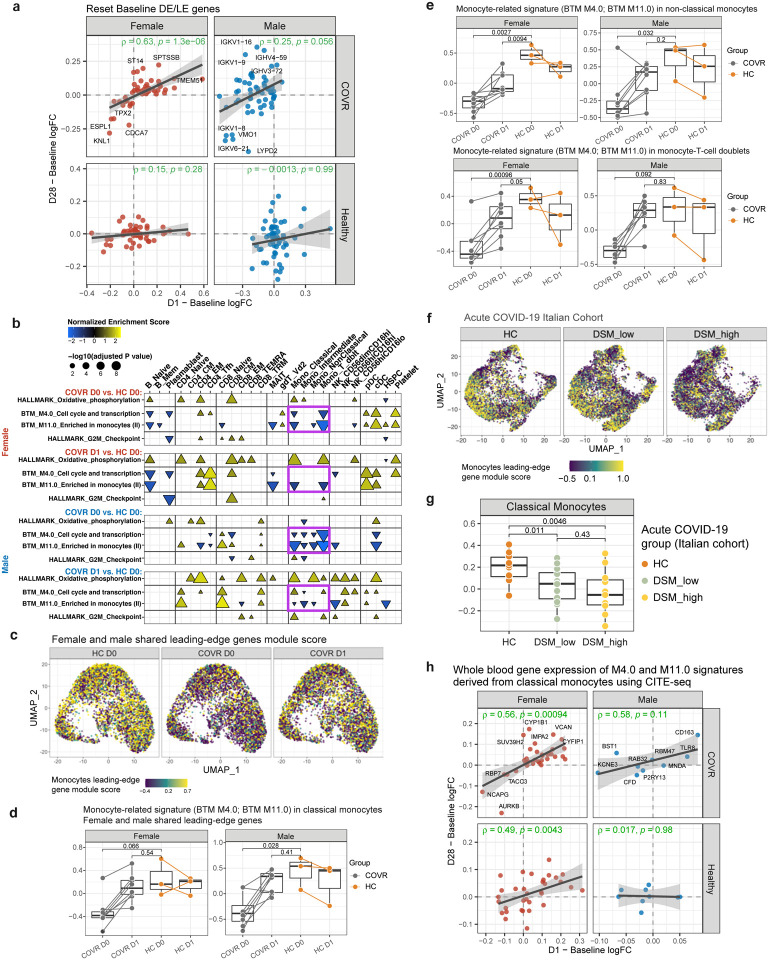
Changes in immune states in COVID-19-recovered individuals following influenza vaccination. **a**, Scatterplots showing the correlation of day 1 (D1; x-axis) and day 28 (D28; y-axis) changes (relative to the pre-vaccination baseline) in whole-blood gene expression for the genes shown in Fig. 4b whose whole-blood expression in COVID-recovered (COVR) moved towards those of healthy (HC) baseline at D1 (see Extended Data Table 8). Genes in the upper right and lower left corners of each scatterplot represent genes whose vaccine-induced shift from baseline are consistent between D1 and D28. Spearman’s rank correlation and p values are shown. **b**, Cell type specific gene expression enrichment plot (derived from CITE-seq data) showing the normalized enrichment scores (GSEA NES) of four selected gene sets (from Fig. 4b) for the indicated group comparisons shown on the left. Positive NES (upward arrow) indicates that gene set scores are higher in the first group than the second group listed in the comparison; negative NES (downward arrow) indicates that gene set scores are higher in the second group than the first group listed in the comparison. The purple boxes highlight the two gene sets (BTM M4.0 and M11.0) enriched for monocyte-related genes. **c**, Similar to Fig. 4c. Transcript-based UMAP visualization of single monocytes (identified by surface proteins) from healthy [day 0 (D0) before vaccination] and COVR males and females at D0 and D1. Cells are colored by the single cell gene module score of the intersection of the male and female “reset” genes (corresponding to the intersection of COVR.MonoSig.F.Mono_Classical and COVR.MonoSig.M.Mono_Classical gene sets as illustrated in Supplemental Information Fig. 3). See Fig. 4c for a similar version that uses the union instead of the intersection of the male and female reset genes. The reset genes were determined using CITE-seq data, essentially correspond to the leading-edge genes (LEGs) of BTM M4.0 and M11.0 that moved towards the healthy baseline by D1 in classical monocytes following influenza vaccination (see also Extended Data Fig. 5b). **d**, Similar to Fig. 4e. Box plots showing the pseudobulk module scores for the same genes as in (c) shown separately for females and males for the indicated sample groups (see Supplemental Information Fig. 3 and Methods). Each dot represents a sample and the D0 and D1 samples from the same individual are connected by a line. P values shown are from t tests of the indicated two group comparisons. Adjusted p-values from the original gene set enrichment tests (GSEA) for the two gene sets (BTM M4.0 and M11.0) are indicated in Extended Data Fig. 5b and Extended Data Table 9B–E. **e**, Similar to (**d**) but comparing the gene module scores for the reset genes in non-classical monocytes (top) and monocyte-T-cell doublets (bottom) shown separately for females and males for the indicated sample groups (see Supplemental Information Fig. 3 and Methods). **f**, Similar to Fig. 4c. Transcript-based UMAP visualization of single monocytes (identified by surface proteins) from the acute COVID-19 CITE-seq dataset in Liu et al (2021). Cells are colored by the single cell gene module score for the LEGs of both BTM M4.0 and M11.0 from males and females (See Supplemental Information Fig. 3a). LEGs are identified from the GSEA analysis comparing COVR samples vs. healthy control at D0 of classical monocytes. Left to right: HC=healthy controls; DSM_low: hospitalized Italian patients with less severe COVID-19 (disease severity metric [DSM] score computed from the earliest timepoint of each subject); DSM_high: those with severe COVID-19. **g**, Box plot comparing the classical monocytes pseudobulk gene module scores using the same LEGs and subject groups as in (**f**). Both males and females are included in all three groups. Each dot represents an acute COVID-19 patient. P values shown are t tests from the indicated two-group comparisons. (GSEA on the acute dataset also indicates significant enrichment of both BTM M4.0 and M11.0 gene sets of COVID-19 vs. HC and DSM-high vs. DSM-low patient comparisons – see Table S4 in *Liu et al*^23^). **h**, Scatterplots showing the correlation between D1(x-axis) and D28 (y-axis) changes (relative to pre-vaccination baseline) in whole-blood gene expression for genes comprising the classical monocyte reset genes derived by CITE-seq, separately for females (COVR.MonoSig.F.Mono_Classical) and males (COVR.MonoSig.M.Mono_Classical). Genes are included in the scatter plots if their D1 whole-blood expression levels in the COVR group moved towards those of the healthy baseline. Genes/dots in the right upper corner of each scatterplot represent stably resolved genes between D1 and D28. Spearman’s rank correlation and p values are shown.

**Figure 4.**
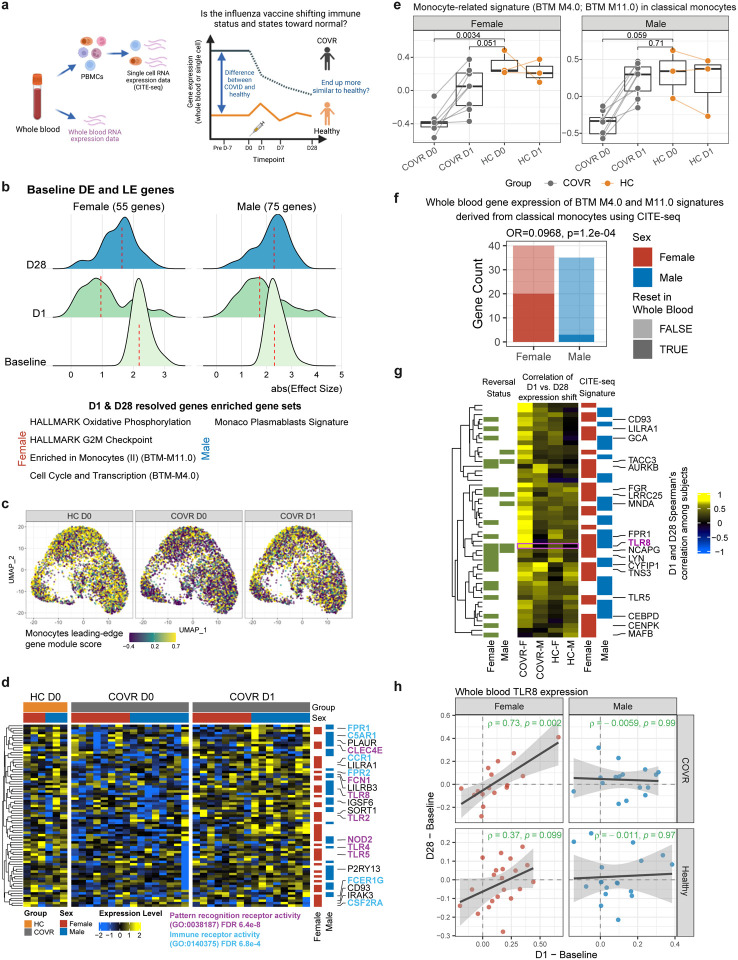
Partial reset of post COVID-19 gene expression imprints by influenza vaccination. **a**, Schematic showing the data types analyzed in this figure (left) and study questions explored (right). D-7 = day −7, D0 = day 0, D1 = day 1, D7 = day 7, D28 = day 28. **b**, (top) Distributions of gene-level absolute difference (measured as model z-scores; see Methods) between COVID-19-recovered (COVR) subjects at each of the indicated timepoints [top to bottom: D28, D1, and the pre-vaccination baseline (baseline includes D-7 and D0)] and the healthy control (HC) baseline for females (HC-F) and males (HC-M), respectively (see Extended Data Table 8). Genes shown are the leading-edge genes (LEGs) from the gene sets listed in Fig. 1e that are also nominally differential expressed (DE) on their own (p-value < 0.05; see Extended Data Table 3). Dashed red vertical lines represent the median of the distribution. (bottom) Enriched gene sets (with more than 5 genes represented in hypergeometric tests) from genes that moved towards healthy baseline at both D1 and D28. **c**, Transcript-based UMAP visualization of single monocytes (identified by surface proteins) from HC (D0 before vaccination), COVR (D0 and D1), both M and F. Cells are colored by the single cell gene module score of the union of the M and F “reset” genes (corresponding to the COVR.MonoSig.F.M.Mono_Classical gene set as illustrated in Supplemental Information Fig. 3). See Extended Data Fig. 5c for a similar version that uses the intersection instead of the union of the M and F reset genes. The reset genes were determined using CITE-seq data, essentially correspond to the LEGs of BTM M4.0 and M11.0 that differed between COVR and HC before vaccination and moved towards the healthy baseline by D1 in classical monocytes following influenza vaccination (see also Extended Data Fig. 5b). **d**, Heatmap showing the pseudobulk expression of the reset genes, separately for M and F, in classical monocytes from CITE-seq data (see Supplemental Information Fig. 3). The reset genes are enriched for the selected Gene Ontology (GO) gene sets shown below (p values from the hypergeometric test). Genes from the GO gene sets are labeled on the right. Samples (columns) are grouped by sex and sample groups (HC at D0, COVR at D0 and COVR at D1), as indicated by the bars above the heatmap. **e**, Box plots showing the pseudobulk module scores of the reset genes in classical monocytes shown separately for F and M for the indicated sample groups (see Supplemental Information Fig. 3 and Methods). Each dot represents a sample and the D0 and D1 samples from the same individual are connected by a line. P values shown are from t tests of the indicated two group comparisons. See also Extended Data Fig. 5b and Extended Data Table 9. **f**, Separately for COVR females (COVR-F) and males (COVR-M), a bar plot showing the proportion of the reset genes (derived from CITE-seq data as detailed in Supplemental Information Fig. 3) whose whole-blood gene expression moved towards the heathy state (defined as the baseline of the HC group) at both D1 and D28 post-vaccination (“reverted”). OR=odds ratio of the likelihood of a gene being reverted between F and M. Pvalue determined by two-tailed Fisher’s exact test. **g**, Spearman’s correlation between D1 and D28 changes relative to baseline in whole-blood gene expression data (Extended Data Table 8) across subjects for the reset genes (rows), separately for F and M (as indicated on the right), in different subject groups (columns). Genes that reverted towards healthy baseline at both D1 and D28 post-vaccination in the COVR subjects are marked (in green) on the left. The D1 vs. D28 correlation for TLR8 is illustrated in (**h**). **h,** Scatterplot showing the correlation of D1 (x-axis) and D28 (y-axis) changes (relative to the pre-vaccination baseline) in whole-blood gene expression of the TLR8 gene within the four indicated groups (COVR-F, COVR-M, HC-F, and HC-M). Spearman’s rank correlation and p values are shown.

Given the potential for longer lasting vaccine “training” effects^36,37^ and that the blood transcriptional responses to the influenza vaccine (Fig. 3b) overlapped with those associated with prior mild COVID-19 (Fig. 1f), we next asked whether influenza vaccination may help “reset” the post-COVID-19 immune states back towards that of healthy controls (pre-vaccination) who never had COVID-19 (“the healthy baseline”, Fig. 4a). As a screen, we first examined genes differentially expressed between the COVID-19-recovered individuals and the matched healthy controls prior to vaccination (Fig. 4b), including the “leading edge” genes from the gene sets associated with prior COVID-19 (Fig. 1f, see Methods). We noted a global “return” towards the healthy baseline in both COVR-F and COVR-M at day 1 after vaccination (Fig. 4b, Extended Data Table 8). Although some of the genes in COVR-M and COVR-F, on average, reverted to their own respective prevaccination states by day 28, there were day 1 changes toward the healthy baseline that persisted through day 28, especially in COVR-F (Extended Data Fig. 5a). These more persistently altered genes in the COVR-F were enriched for cell cycle, oxidative phosphorylation, and monocyte related genes (Fig. 4b). Thus, the early inflammatory response to the influenza vaccine might have led to the resolution of some of the previously stable differences between COVR-F and HC-F; while similar changes were also detectable in COVID-19 recovered males by day 1, on average they returned to their own (COVR-M) pre-vaccination baseline state by day 28 following vaccination.

Given that whole blood transcriptomic changes can result from a mix of cell composition and cell intrinsic transcriptional changes, we next used CITE-seq (Cellular Indexing of Transcriptomes and Epitopes by Sequencing;^38^) to assess the cellular source of the day 1 transcriptional changes. CITE-seq simultaneously profiled surface proteins and transcriptomes of single PBMCs from COVID-19-recovered subjects and matching healthy controls (Fig. 4a). We clustered single cells and annotated the resulting clusters/subsets using surface protein expression profiles (see Methods). Separately in males and females we assessed the cell subsets in which the gene sets with whole blood transcriptomic changes (bottom of Fig. 4b; see Methods) tend to be differentially expressed between COVID-19-recovered subjects and healthy controls prior to vaccination and then, in the recovered subjects, moved towards the baseline (day 0) state of the healthy controls on day 1 after vaccination. This revealed that the two monocyte-related gene sets [blood transcriptomic modules M4.0 and M11.0;^39^] were indeed altered (i.e., less depressed compared to healthy on day 1) in monocytes by vaccination in both COVR-M and COVR-F, especially in classical monocytes and monocyte-T cell doublets (Extended Data Fig. 5b). Thus, monocytes were a major source of the reset signal we detected using bulk gene expression data above (Fig. 4b).

By using the single cell data separately in females and males, we further pinpointed the genes (the “reset genes”) from the two gene sets (M4.0 and M11.0) that drove the reversal towards the healthy state within classical monocytes by day 1 following vaccination (see Methods, Supplementary Information Fig. 3). UMAP and heatmap visualizations confirmed that the female and male reset genes (or their union or intersection) had lower expression in the monocytes of COVID-19-recovered subjects before vaccination, but their expression was then elevated by day 1 and moved towards the healthy baseline following influenza vaccination (Fig. 4c–e, Extended Data Fig. 5c, d); these trends were similar across the major monocyte subsets, including non-classical monocytes (Extended Data Fig. 5e) and subclusters of single monocytes defined by mRNA profiles (data not shown). Thus, these vaccine-induced changes by day 1 after vaccination in COVID-19-recovered subjects were unlikely driven by changes in monocyte composition alone (e.g., new monocytes emerging from the bone marrow) but intrinsic to most if not all circulating monocytes.

**Extended Data Figure 6.**
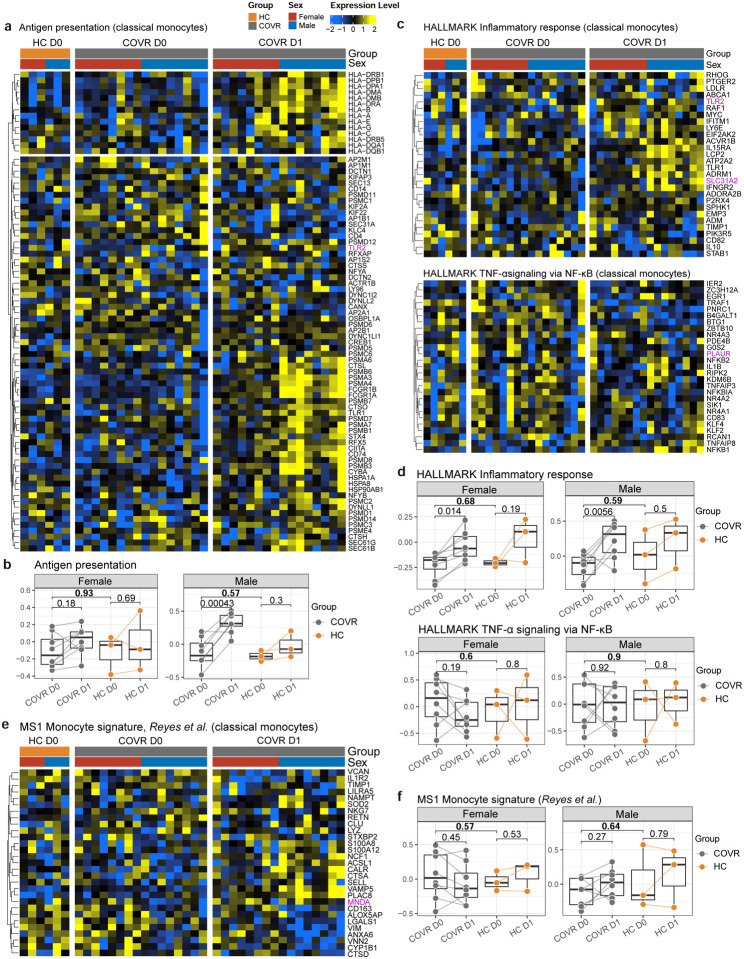
Gene expression profile of antigen presentation, NF-κB/inflammatory, and monocytic myeloid-derived suppressor cell (MDSC) related signatures in classical monocytes **a,** Heatmap showing the pseudobulk expression of the leading-edge genes (LEGs) from antigen presentation related gene sets, separately for male (M) and female (F), in classical monocytes from CITE-seq data. The LEGs are from the acute COVID-19 vs. healthy control (HC) GSEA analysis in^23^, which showed that genes in the antigen presentation gene sets – KEGG_Antigen processing and presentation, Reactome_Antigen processing-Cross presentation, Reactome_MHC class II antigen presentation – tend to be lower in COVID-19. Samples (columns) are grouped by sex and sample groups HC at day 0 (D0), COVID-19-recovered (COVR) at D0 and COVR at day 1 (D1), as indicated by the bars above the heatmap. Gene names are shown on the right; those in purple correspond to genes also in the “reset” signature. **b,** Box plots showing the pseudobulk module scores of the LEGs as in **(a)** in classical monocytes shown separately for F and M for the indicated sample groups. Each dot represents a sample and the D0 and D1 samples from the same individual are connected by a line. P values shown are from t tests of the indicated two group comparisons. **c,** Similar to **(a)**, but showing the LEGs of HALLMARK Inflammatory response (top) and HALLMARK TNF-α signaling via NF-κB (bottom) gene sets; LEGs derived from the acute COVID-19 vs. HC GSEA analysis in *Liu et al*^23^. **d,** Similar to **(b)**, but showing the pseudobulk module scores calculated from the LEGs of HALLMARK Inflammatory response (top) and HALLMARK TNF-α signaling via NF-κB (bottom) as in **(c).** **e,** Similar to **(a)**, but showing the genes of MSDC/MS1 monocyte signature from *Reyes et al*^42^. **f,** Similar to **(b)**, but showing the pseudobulk module scores calculated from the genes of MSDC/MS1 monocyte signature as in **(e).**

The reset genes are enriched for pattern recognition/immune receptor and innate defense genes, including those encoding Toll-like receptors (TLR2, TLR5, and TLR8), the peptidoglycan recognizing receptor NOD2, the high affinity IgE FC receptor FCER1G, and formyl peptide receptors (Fig. 4d). We next wondered whether these monocyte alterations seen in the COVID-19-recovered (but otherwise healthy) subjects months after mild COVID-19 could be linked to gene expression changes seen in acute disease. Using a previously published CITE-seq dataset we generated from a hospitalized, predominantly older and male-biased COVID-19 cohort from Italy^23^, we noted that within the classical monocytes, the average expression of the reset genes was significantly lower in COVID-19 patients than healthy controls and negatively associated with disease severity [Extended Data Fig. 5f, g; similar for union or intersection of the male and female reset genes (data not shown)]. Thus, the gene expression changes in the monocytes of COVID-19-recovered subjects could have originated from and persisted since the early response to the infection. Several studies have reported the increase of several (potentially overlapping) types of altered monocytes in acute COVID-19, including those with lower antigen presentation, depressed NF-kB/inflammation, or myeloid-derived suppressor cell (MDSC)-like phenotypes^23,40–44^. However, none of them were significantly different in the pre-vaccination monocytes of COVID-19-recovered subjects compared to HCs in our cohort (Extended Data Fig. 6a–f), suggesting that our reset gene signature is distinct from these monocyte phenotypes found in acute disease. Consistent with the observations above (Fig. 3b–e), these single cell data also revealed that COVR-M had more robust antigen presentation transcriptional responses than COVR-F and HCs on day 1 following influenza vaccination (Extended Data Fig. 6a, b). Together, CITE-seq analysis revealed that the early (day 1) response to influenza vaccination elevated a set of previously (i.e., before vaccination) depressed innate immune receptor/defense genes in the monocytes of COVID-19-recovered subjects.

We further evaluated whether the female and male monocyte reset genes might have persisted to day 28 using the bulk, whole blood expression data in females and males, respectively (Fig. 4f, Extended Data Table 8). We first noticed that a larger fraction of the female reset genes demonstrated reversal in average expression towards the healthy baseline state by day 28 compared to the male reset genes in the bulk expression data (Fig. 4f). Interestingly, in COVR-F the day 1 changes (mostly increases in gene expression, as expected, given the depressed state of these genes in monocytes) for most genes tended to be temporally stable and persisted to day 28 – i.e., the fold changes between day 1 and day 0 are positively correlated with those between day 28 and day 0; this was less evident in HC-F and in COVR-M or HC-M (Fig. 4g, h, Extended Data Fig. 5h, Extended Data Table 8). This result is consistent with our earlier observation above (Fig. 4b) that some of the early reversal genes, determined using bulk expression data, persisted to day 28 more in COVR-F. Thus, while the reset genes were impacted in monocytes by day 1 following influenza vaccination in both COVID-19-recovered males and females, persistence to day 28 was more evident in females. Together, these results identified a depressed innate defense gene expression signature in monocytes associated with prior mild COVID-19 in both sexes and suggest that the early inflammatory responses to influenza vaccination could help revert this immune status back towards the healthy state, particularly in COVID-19-recovered females.

## Discussion

While both acute and long-term immune perturbations in hospitalized COVID-19 patients have been reported^21,44–49^, less is known regarding healthy recovered individuals with prior mild, non-hospitalized SARS-CoV-2 infection months after acute illness. Furthermore, most studies of post-COVID-19 have focused on adaptive and antigen-specific immunity. Here we reveal that prior mild, non-hospitalized COVID-19 in otherwise healthy individuals is associated with sex-specific immune imprints beyond SARS-CoV-2 specific immunity, some of which only become apparent after heterologous challenge via influenza vaccination (i.e., a vaccine that is antigenically distinct from SARS-CoV-2). Thus, COVID-19 has the potential to impact the response to future immunological perturbations long after acute disease and convalescence. This is of public health importance given that the majority of the more than 370 million global SARS-CoV-2 infections have been mild and not required hospitalization^50^. The few studies of convalescent, mild COVID-19 have included patients with multiple medical co-morbidities, relatively small sample sizes, and did not evaluate sex-specific effects^51–53^. To our knowledge, ours is the first study to reveal sex-specific molecular and cellular immune imprints and future immune response differences associated with prior mild COVID-19 in otherwise healthy individuals, particularly those without confounding comorbidities such as autoimmunity or immunodeficiency. Given the heightened innate responses, increased interferon production, and elevated antibody generation following influenza vaccination in COVID-19-recovered males, our study demonstrates that an *in vivo* heterologous vaccine challenge together with systems biology analyses can help elucidate molecular and cellular immunological differences in post-COVID-19 patients.

Our findings are consistent with the sex dimorphic nature of acute responses to SARS-CoV-2 and other immune challenges^11–16,18,19,54^. Females are generally more susceptible to autoimmunity and tend to mount heightened inflammatory responses to infections and vaccines^55^; it was therefore surprising to find the qualitative opposite here in which COVID-19-recovered males were found to have a more “activated” immune status at baseline and stronger innate and adaptive responses to influenza vaccination. While some of these might be attributable to differences in acute disease severity (e.g., males tended to have more severe disease than females), it is not clear how that might have manifested in our mild, non-hospitalized patients as neither the self-reported duration of acute illness nor antibody titers against SARS-CoV-2 were different between COVR-M and COVR-F (data not shown), which together suggest that our observations are independent of severity or immune response quality during acute disease. Persistent immune state changes (over months) in patients with “long COVID” have recently been reported^45,56^, but most of the individuals in our study reported no or at worst minor post-COVID-19 sequelae. Thus, immunological modifications with functional consequences can still be present after clinically resolved, mild COVID-19. Although our study found heterologous vaccine response benefit in COVR-M (e.g., elevated influenza vaccine titers), the impact of prior mild COVID-19 on other perturbations such as non-SARS-CoV-2 respiratory infections remains to be determined. For example, airway neutrophil inflammation before respiratory syncytial virus exposure is associated with symptomatic outcomes^57^. As future work it could also be informative to assess whether some of the sex-specific imprints, including the differences in heterologous vaccination responses identified here, are associated with clinical sequelae present in those with “long COVID”^1^.

The sex-specific post-vaccination cellular and molecular dynamics observed in this study (Fig. 3) suggest that the more “primed” baseline immune states in COVR-M (Fig. 1f–h) could have helped establish the more robust IFN, plasmablast, and antibody responses on days 1, 7, and 28, respectively, following influenza vaccination, which is antigenically distinct from SARS-CoV-2. These observations are consistent with findings that the heterologous (non-antigen-specific) effects of vaccination (e.g., BCG) can be sex-specific^58^. Interestingly, a qualitatively similar innate “priming” effect has also been observed in repeated homologous vaccination, such as increased innate responses following the second dose of the Pfizer-BioNTech COVID-19 vaccine or the AS01-adjuvanted hepatitis B vaccine compared to the first dose^59,60^. Although these particular homologous (repeated dosing) vaccine-induced responses were not sex-specific and the second dose was given only 3–4 weeks after the first (compared to the months between mild COVID-19 and influenza vaccination in our study), these data support the hypothesis that similar to a first vaccine/inflammatory exposure, prior mild SARS-CoV-2 infection might have acted through certain immune pathways to prime a stronger early IFN and subsequent plasmablast responses in COVR-M after influenza vaccination.

Changes in the transcriptional and epigenetic profiles of peripheral monocytes have been described in both acute and convalescent COVID-19 patients with moderate-to-severe disease, but few included patients months out from infection^41,43,47,49,61^. These previously described changes during acute disease include the depressed inflammation/antigen-presentation transcriptional phenotypes that are, as shown above, distinct from our reset signature detected months post COVID-19 (Extended Data Fig. 6). This monocyte imprint involving transcriptionally depressed innate defense/receptor genes is consistent with the notion of trained immunity^36^. However, our signature likely reflects different biology than the “poised”, trained monocytes (based on epigenetic and *in vitro* stimulation studies) found in an earlier study of seven COVID-19-recovered patients, probably because those were hospitalized patients with more severe acute disease (e.g., most had pneumonia) and the time since discharge was relatively short (~4–12 weeks)^49^. The finding that the monocyte imprint we detected was partially reversible by seasonal influenza vaccination suggests that in addition to providing antigen-specific protection, vaccines could help reset certain immune cell states in an antigen-agnostic manner. Whether that was achieved through reprogramming of certain myeloid progenitor cells in the bone marrow remains to be dissected, as do mechanisms on how COVID-19 can train immune cell statuses, and how training and vaccine-induced reversal depend on parameters such as sex, clinical factors such as acute disease severity, and age.

**Limitations of this study** include most study subjects were younger than 65 and thus these findings may not apply to the elderly, an important population of COVID-19 recoverees. Additionally, our findings are largely associative in nature and the study design does not allow the linking of acute response phenotypes to the long-term imprints in the same individuals. Some of the imprints we considered as stable given lack of association with TSD may still be evolving slowly (or could be limited by statistical power for detecting association with TSD). And while there was no clear difference in disease severity or duration between the COVID-19-recovered males and females in our study (and no subjects were hospitalized), it is possible that our sex-specific findings reflect unappreciated clinical factors. It is possible that some of the post-vaccination reversal towards the healthy, pre-vaccination state by day 28 may also in part be due to ongoing disease resolution. However, this is unlikely the case for the vaccine-induced elevation in the expression of the reset genes towards the healthy state because those changes were clearly detectable on day 1 after vaccination and persisted through day 28, especially in females, indicating that this reversal was driven (or at least accelerated) by vaccination and could not be attributed to the “natural” resolution process alone. While it would be informative to further assess our findings in follow up cohorts, given our observation that vaccination could perturb some of the immune imprints associated with prior mild COVID-19, identification and recruitment of a sufficient number of individuals who have not had the influenza or COVID-19 vaccines since their COVID-19 disease would be impractical. The functional and clinical implications of the vaccine-induced reversal of the reset gene signature in monocytes remain to be determined. Despite these limitations, our work provides conceptual advances regarding how even mild viral infections can stably shape human immune statuses and functions long-term after acute illness, thus establishing new antigen agnostic baseline set point with potential impacts on future responses^62^, and in turn, how heterologous vaccination can reveal such imprints and potentially help reset the immune system back towards the state before SARS-CoV-2 infection.

## Methods

### Patient population and sample collection

Subjects at least 18 years of age were recruited from the local area (Maryland, Virginia, and the District of Columbia) and enrolled on National Institutes of Health (NIH) protocol 19-I-0126 (Systems analyses of the immune response to the seasonal influenza vaccine). Exclusion criteria included obesity (BMI ≥ 30); history of any autoimmune, autoinflammatory or immunodeficiency disease; history of any vaccine within the past 30 days (live attenuated) or 14 days (non-live attenuated); history of any experimental vaccine; history of a parasitic, amebic, fungal, or mycobacterial infection in the past year; or current infection.

Samples were collected on subjects from three groups: 1) those with a prior history of symptomatic SARS-CoV-2 infection (defined as a history positive nasal PCR test and positive Food and Drug Administration (FDA) Emergency Use Authorization (EUA) SARS-CoV-2 antibody test at the time of protocol screening), 2) those with a history of asymptomatic SARS-CoV-2 infection (defined as a positive FDA EUA SARS-CoV-2 antibody test at the time of protocol exam but no history of COVID-like symptoms), and 3) individuals with no history of SARS-CoV-2 infection (defined as a negative FDA EUA SARS-CoV-2 antibody test at the time of the protocol screening).

Blood for PBMCs, serum, whole blood RNA [Tempus^™^ Blood RNA Tube (Thermo Fisher Scientific, Waltham, MA)], complete blood count with differential (CBC) and lymphocyte phenotyping was collected at each of the following timepoints relative to seasonal influenza vaccination (day 0): days −7, 0, 1, 7, 14, 28, 70, 100. Optional stool was collected at days 0, 28 and 100. Subjects were provided with Cardinal Health Stool Collection kits (Cardinal Health, Dublin, OH) and Styrofoam storage containers with ice packs to collect stool samples at home and return in person to the NIH. Following day 100, subjects had the option to continue to provide monthly blood samples for PBMCs, serum, whole blood RNA, CBC with differential and lymphocyte phenotyping through August 2021.

Study data were collected and managed using REDCap electronic data capture tools hosted at the NIH^63,64^. REDCap (Research Electronic Data Capture) is a secure, web-based software platform designed to support data capture for research studies, providing 1) an intuitive interface for validated data capture; 2) audit trails for tracking data manipulation and export procedures; 3) automated export procedures for seamless data downloads to common statistical packages; and 4) procedures for data integration and interoperability with external sources. REDCap electronic questionnaires were utilized to collect information from participants via two separate IRB-approved surveys. A survey to evaluate vaccine-related adverse events or symptoms was administered on study days 1 and 7 and a separate survey to evaluate for any health changes or new medications was administered at every visit starting on Day 0. Surveys were sent via email to the participants and responses were transferred from the REDCap system to the NIH Clinical Research Information Management System (CRIMSON) system by the study team.

### Influenza vaccination

Subjects between ages 18 – 64 years were administered the Flucelvax Quadrivalent seasonal influenza vaccine (2020–2021; Seqirus Inc, Summit, NJ). Subjects 65 years of age and older were administered the high-dose Fluzone Quadrivalent seasonal influenza vaccine (2020–2021; Sanofi Pasteur Inc, Swiftwater, PA).

### Influenza microneutralization titers

Virus-neutralizing titers of pre- and post-vaccination sera were determined in a microneutralization assay based on the methods of the pandemic influenza reference laboratories of the Centers for Disease Control and Prevention (CDC) using low pathogenicity vaccine viruses and MDCK cells. The X-179A virus is a 5:3 reassortant vaccine containing the HA, NA, and PB1 genes from A/California/07/2009 (H1N1pdm09) and the 5 other genes from A/PR/8/34 were donated by the high growth virus NYMC X-157. Immune sera were also tested for neutralization titers of the seasonal vaccine strains H1N1 A/Brisbane/59/07, H3N2 A/Uruguay/716/07, and B/Brisbane/60/2001. Internal controls in all assays were sheep sera generated against the corresponding strains at the Center for Biologics Evaluation and Research, FDA, Bethesda, MD. All individual sera were serially diluted (2-fold dilutions starting at 1:10) and were assayed against 100 TCID50 of each strain in duplicates in 96-well plates (1:1 mixtures). The titers represent the highest dilution that completely suppressed virus replication.

### SARS-CoV-2 pseudovirus production and neutralization assay^65–67^

Human codon-optimized cDNA encoding SARS-CoV-2 S glycoprotein (NC_045512) was cloned into eukaryotic cell expression vector pcDNA 3.1 between the *BamH*I and *Xho*I sites. Pseudovirions were produced by co-transfection of Lenti-X 293T cells with psPAX2(gag/pol), pTrip-luc lentiviral vector and pcDNA 3.1 SARS-CoV-2-spike-deltaC19, using Lipofectamine 3000. The supernatants were harvested at 48h post transfection and filtered through 0.45-μm membranes and titrated using 293T-ACE2 cells (HEK293T cells that express ACE2 protein). The following reagent was obtained through BEI Resources, NIAID, NIH: Human Embryonic Kidney Cells (HEK-293T) Expressing Human Angiotensin-Converting Enzyme 2, HEK-293T-hACE2 Cell Line, NR-52511.

For the neutralization assay, 50 μL of SARS-CoV-2 S pseudovirions were pre-incubated with an equal volume of varying dilutions of serum at room temperature for 1 h, then virus-antibody mixtures were added to 293T-ACE2 cells in a 96-well plate. After 3 h incubation, the inoculum was replaced with fresh medium. After 24 hours, cells were lysed and luciferase activity was measured. Controls included cell only control, virus without any antibody control and positive control sera.

### SPR based antibody binding kinetics of human serum^68–70^

Steady-state equilibrium binding of serum was monitored at 25°C using a ProteOn surface plasmon resonance (BioRad). The purified recombinant SARS-CoV-2 or other proteins were captured to a Ni-NTA sensor chip (BioRad, Catalog number: 176–5031) with 200 resonance units (RU) in the test flow channels. The protein density on the chip was optimized such as to measure monovalent interactions independent of the antibody isotype. Serial dilutions (10-, 30- and 90-fold) of freshly prepared sample in BSA-PBST buffer (PBS pH 7.4 buffer with Tween-20 and BSA) were injected at a flow rate of 50 μL/min (120 sec contact duration) for association, and disassociation was performed over a 600-second interval. Responses from the protein surface were corrected for the response from a mock surface and for responses from a buffer-only injection. Total antibody binding was calculated with BioRad ProteOn manager software (version 3.1). All SPR experiments were performed twice, and the researchers performing the assay were blinded to sample identity. In these optimized SPR conditions, the variation for each sample in duplicate SPR runs was <5%. The maximum resonance units (Max RU) data shown in the figures were the RU signal for the 10-fold diluted serum sample.

### PBMC isolation

PBMC samples were isolated from blood collected in Vacutainer EDTA tubes (generic lab supplier) using the SepMate^™^−50 tubes (STEMCELL Technologies, Cambridge, MA) with following modifications to the manufacturer’s protocol: The blood samples were diluted 1:1 with room temperate PBS and mixed by pipetting. The diluted blood was layered on top of 15ml Cytiva^™^ Ficoll^™^ PAQUE-Plus (Cytiva Life Sciences, Marlborough, MA) layer in SepMate. The SepMate tubes were spun at 1200 g for 10 mins with brake set to 5 at room temperature. Following the spin, the top plasma layer was removed as much as possible without disturbing the PBMC layer. If there were any cells stuck on the wall of the tube, then they were gently scraped from the wall with pipette, so they can be resuspended with rest of the cells. The cells were poured from SepMate in to a 50ml conical tube. The tubes containing cells were filled up to 50ml with cold wash buffer (PBS with 2% FBS) and mixed by inverting. The tubes were spun at 300 g for 10 mins with brake set to 5 at room temperature. After the spin, the supernatant was removed without disturbing the cell pellet. After resuspending the pellet with cold wash buffer, the cells were counted using the Guava® Muse® Cell Analyzer (Luminex Corporation, Austin, TX). The tubes were again spun at 300 g for 10 mins with brake set to 5 at room temperature. The supernatant was removed without disturbing the cell pellet.

Based on the cell count, 6 – 10 million PBMC were frozen per vial for each sample. Since the cells were counted prior to the last spin, a 50% cell loss was assumed and accounted for in the calculations from cell count. The cell pellet was resuspended with n*600μl (n = number of PBMC vials to be frozen) freezing media (RPMI with 10% FBS) by gentle pipetting. After freezing media, n*600μl DMSO freeze (FBS with 15% DMSO) was added drop-by-drop while gently shaking the tube. In other words, for each vial of PBMC that was to be frozen, 600μl of freezing media and 600μl of DMSO freeze was added, bringing the total volume for each vial to 1.2ml. The solution was gently mixed by pipetting before transferring 1.2ml cell solution to each 1.8ml cryovial (general lab supplier). The cell vials were placed in CoolCell Containers (Thomas Scientific, Swedesboro, NJ) and the container was placed in a −80°C freezer. After at least 4 hours, the PBMC vials were transferred to liquid nitrogen.

### RNA isolation

Blood was drawn directly into the Tempus^™^ Blood RNA Tube (Thermo Fisher Scientific, Waltham, MA) according to manufacturer’s protocol. Two Tempus tubes were collected at each study timepoint. The blood sample from each Tempus tube was aliquoted in to two 4.5mL cryovials (General lab supplier). These cryovials were directly stored at −80°C. The RNA samples were isolated in groups of 12–22 samples per batch based on careful batching prior to isolation to reduce confounding factors due to age, gender, and patient group.

RNA was isolated from tempus blood using the QIAsymphony RNA Kit (Qiagen, Gaithersburg, MD) on QIAsymphony SP instrument (Qiagen, Gaithersburg, MD). Blood samples were thawed on ice before each sample was transferred to a 50ml conical tube. The total volume of the sample was brought to 12ml by adding 1x PBS. The tubes were vortexed at full speed for 30 seconds, followed by centrifugation at 3500 g for 1 hour at 4°C. After centrifugation, the supernatant from the tubes was decanted and tubes were placed upside down on clean paper towels for 2 minutes to allow residual liquid to drain. To resuspend the pellet, 800μl of RLT+ buffer was added to the bottom of each tube and vortexed for few seconds. All 800μl of each sample was transferred to 2ml screw cap tubes (Sarstedt, Nümbrecht, Germany). The tubes were placed into #3b adapters (Qiagen, Gaithersburg, MD) to be loaded on to the QIAsymphony.

On the QIAsymphony, RNA CT 800 protocol was selected and used for RNA isolation. The instrument was set up according to the manufacturer’s protocol and the elution volume for RNA samples was set to 100μl. The final volume of the eluted RNA samples ranged from 65 – 95 μl.

RNA yields were determined using Qubit RNA BR kit or Qubit RNA HS kit (Thermo Fisher Scientific, Waltham, MA) based on the yield. RNA RIN numbers were measured using RNA ScreenTape (Agilent Technologies, Santa Clara, CA). The average RIN was 8.3 and average yield was 81.3 ng/μl for the RNA samples.

### RNA-seq

RNA-seq libraries were prepared manually using Universal Plus mRNA-Seq with NuQuant, Human Globin AnyDeplete (Tecan Genomics, Redwood City, CA) according to manufacturer’s protocol. For each sample, 500ng of total RNA was used to isolate mRNA via poly(A) selection. Captured mRNA was washed, fragmented, and primed with the mix of random and oligo(dT) primers. After cDNA synthesis, ends were repaired and ligated with Unique Dual Index (UDI) adaptor pairs. Unwanted abundant transcripts from rRNA, mtRNA and globin were removed using AnyDeplete module. Remaining library was amplified by 14 cycles of PCR and purified with AMPure XP reagent (Beckman Coulter, Indianapolis, IN).

Library concentration was determined by Quant-iT^™^ PicoGreen^™^ dsDNA Assay kit (Thermo Fisher Scientific, Waltham, MA) on BioTek Synergy H1 plate reader (BioTek Instruments, Winooski, VT) using 2 ul sample. Library size distribution was determined using D1000 ScreenTape (Agilent Technologies, Santa Clara, CA) on 4200 TapeStation System (Agilent Technologies, Santa Clara, CA). Thirty-two samples were randomly selected from each plate to measure the library size distribution. To determine fragment size, the region on the electropherogram was set from 200 bp to 700 bp. An average of the fragment sizes was used for the rest of libraries to calculate molarity.

To create a balanced pool for sequencing, all libraries from one plate were diluted to the same molar concentration by the QIAgility liquid handling robot (Qiagen, Gaithersburg, MD) and equal volumes of normalized samples were pooled. Ninety-six samples were pooled from each plate on Plates 1–4 and 35 samples were pooled from Plate 5. For an accurate quantification of the pooled libraries, a qPCR was performed using KAPA Library Quantification Kit (Roche, Wilmington, MA).

All libraries were sequenced on the NovaSeq 6000 instrument (Illumina, San Diego, CA) at Center for Cancer Research Sequencing Facility, National Cancer Institute. The libraries pooled from Plates 1–4 were sequenced using one NovaSeq 6000 S4 Reagent Kit (200 cycles) and NovaSeq XP 4-Lane Kit (Illumina, San Diego, CA) with sequencing parameter as 100 bp paired-end reads. The library pool from Plate 5 was sequenced using a NovaSeq 6000 SP Reagent Kit (300 cycles; Illumina, San Diego, CA) with 150 bp paired-end reads as sequencing parameter.

Additionally, after quality control, 11 samples were re-sequenced as Plate 6 on a NextSeq 500 instrument using a NovaSeq 6000 S4 Reagent Kit (200 cycles) with sequencing parameter as 100 bp paired-end reads. Technical replicates were placed on each plate to control for plate variability.

### Serum isolation

Serum was collected directly in Serum Separator Tubes, and allowed to clot at room temperature for a minimum of 30 minutes. Within two hours of blood collection, the tubes were spun at 1800 g for 10 minutes at room temperature. The top (serum) layer was removed via pipette and stored in individual vials at −80°C.

### Complete Blood Counts and lymphocyte phenotyping

Subjects had standard complete blood counts with differential (CBCs) performed at the NIH Clinical Center in the Department of Laboratory Medicine. Lymphocyte (T cell, B cell, NK cell) flow cytometry quantification was performed using the BD FACSCanto^™^ II flow cytometer (BD Biosciences, Franklin Lakes, NJ).

### Flow cytometry

#### a) B cell panel including influenza HA probes

Thawed PBMC were washed in RPMI culture medium containing 50U/ml benzonase nuclease and then washed by PBS. Cells were incubated with LIVE/DEAD Fixable Blue Dye (Life Technologies, Carlsbad, CA), which was used to exclude dead cells from analysis. Cells were incubated with fluorochrome-conjugated HAs for influenza B (B/Washington/02/2019 and B/Phuket/3073/2013 combined on the same fluorochrome), and Influenza A H1 (A/Hawaii/70/2019) and H3 (A/Hongkong/2671/2019) and fluorochrome-conjugated antibodies against IgM, IgA, CD21, CD85J, FCRL5, CD20, IgG, CD38, CD14, CD56, CD3, CD27, CD71, CD19, IgD for 30 min at 4 C in the dark. The dyes and detailed information of antibodies in the panel (Sarah Andrews, Vaccine Research Center, National Institute of Allergy and Infectious Diseases, National Institutes of Health) are summarized in Table 1. After incubation with antibodies for 30 minutes, cells were washed two times with FACS buffer (0.1%BSA/PBS (pH7.4)) and fixed in 1% paraformaldehyde. Five million cells were acquired on Cytek Aurora spectral cytometer (Cytek Biosciences, Fremont, CA). Data were analyzed with FlowJo software version 10 (BD Biosciences).

#### b) Phenotyping panel

Thawed PBMC were washed in RPMI culture medium containing 50U/ml benzonase nuclease and then washed by PBS. Cells were incubated with LIVE/DEAD Fixable Blue Dye (Life Technologies, Carlsbad, CA), which was used to exclude dead cells from analysis. Cells were washed in FACS staining buffer (1 × phosphate-buffered saline, 0.5% fetal calf serum, 0.5% normal mouse serum, and 0.02% NaN_3_) and incubated with Human Fc block reagent (BD bioscience #564220) at room temperature for 5 min. Cells stained at room temperature for 10 minutes in the dark with fluorochrome-conjugated antibodies against CCR7, CCR6, CXCR5, CXCR3 and TCRgd. Then, stained with fluorochrome-conjugated antibodies against CD45RA, CD16, CD11c, CD56, CD8, CD123, CD161, IgD, CD3, CD20, IgM, IgG, CD28, PD-1, CD141, CD57, CD45, CD25, CD4, CD24, CD95, CD27, CD1c, CD127, HLA-DR, CD38, ICOS, CD21, CD19, CD14 at room temperature for 30 minutes in the dark. Cells were washed two times with FACS staining buffer (1 × phosphate-buffered saline, 0.5% fetal calf serum, 0.5% normal mouse serum, and 0.02% NaN_3_) and fixed in 1% paraformaldehyde. Table 2 shows all the clones and information of antibodies used in the phenotyping panel. A million PBMC were acquired by using Cytek Aurora spectral cytometer (Cytek Biosciences, Fremont, CA). The frequency of major populations was analyzed using with FlowJo^™^ software version 10 (BD Biosciences) based on previously described manual gating strategies^71–73^.

### Data processing and transformation

#### Bulk RNA-seq data processing

Sequencing reads from Plate 5 were adaptor- and quality-trimmed to 100 bp using Trimmomatic^74^ to match the read length of the other plates (resulting reads with less than 100 bp were discarded). Reads were then aligned to the human genome hg38 using the STAR aligner. Duplicate reads from PCR amplification were removed based on Unique Molecular Identifiers (UMI). Gene expression quantification was performed using the featureCounts^75^ function from Subread package. Samples with less than 5 million assigned reads were resequenced and replaced. Reads were normalized and log transformed using *limma voom*^76^. Lowly expressed genes, defined as having fewer than five samples with > 0.5 counts per million reads, were removed. Pre-vaccination (days −7 and 0) samples from the same healthy control (HC) subjects were considered as replicates and were used to estimate latent technical factors by the RUVs function from the SeqRUV^77^ R package. Four latent variables were included to derive normalized gene expression values used for visualization and when specifically noted. Variable genes based on intra-subject variability of pre-vaccination samples in the HCs and across technical replicates were filtered out, resulting in a total of 10017 remaining genes for downstream analyses.

#### OLINK serum proteomics

Missing values were imputed using k-nearest neighbors approach with k=10. For each sample, probes targeting the same protein were averaged.

#### Cytek flow cytometry

Cell frequencies were generated by converting cell counts as fraction of live cells or lymphocytes as specified. The frequency data were log2 transformed for linear modeling. For populations with zero counts in any of the samples, an offset equaling to half of the smallest non-zero value was added across samples.

#### CBC with diff and TBNK

Both absolute and relative counts were log2 transformed for linear modeling. For parameters with zero values in any of the samples, an offset equaling to half of the smallest non-zero value was added across samples.

#### Serology

Influenza antibody titer and surface plasmon resonance (SPR)-based antibody affinity data were log2 transformed for linear modeling. Maximum titer (shown in Fig. 2c) was calculated by normalizing titer levels across all samples from both day 0 and day 28 individually for each of the four strains followed by taking the maximum standardized titer for each sample. Maximum fold change (MFC; shown in Extended Data Fig. 3c) was defined in^78^ and as the maximum of the day 28 over day 0 fold change across all titers, based on normalized values.

#### Baseline differential expression analysis

Using the dream^79^ function in the variancePartition R package, mixed-effects models were applied to determine differential levels of analytes (i.e. whole-blood gene expression, serum proteins, cell frequencies, flu titer and SPR, and hematological parameters) between COVID-recovered and HC subjects in a sex-specific manner as follows:

$${}^{\sim }0+\text{group:sex}+\text{age}+\text{race}+\text{batch.effects}+(1|\text{subject.id) }$$

Batch effect-related covariates were added to specific models depending on the assay type. For bulk RNA-seq, these include the four latent technical factors (see Bulk RNA-seq data processing) and the timepoint-matched % neutrophils parameter from the CBC panel. For the Cytek and Olink platforms, sampling batch/plate was included as covariates. In addition to day 0, available samples from day −7 (in RNA-seq and CBC panel), were included as baseline replicates in the modeling.

Sex-specific group differences were computed from the contrasts covid.Female – healthy.Female and covid.Male – healthy.Male. Overall COVID vs. HC difference was determined by combining the two contrasts, i.e. (covid.Female – healthy.Female)/2 + (covid.Male – healthy.Male)/2. Sex difference linked to SARS-CoV2 infection was derived from the contrast (covid.male – covid.female) – (healthy.male – healthy.female) to account for normal differences between males and females. P values were adjusted for multiple testing within each assay type and contrast combination using the Benjamini-Hochberg (BH) method (Benjamini and Hochberg, 1995).

#### Association with time since COVID-19 diagnosis

To evaluate whether any of the differences detected at baseline had stabilized or might still be resolving, a linear model was used to test the association of relevant parameters with the time since COVID-19 diagnosis (TSD) among COVID-recovered subjects:

$${}^{\sim }0+\text{sex}+\text{sex}:\text{scale}(\text{TSD})+\text{age}+\text{race}+(1|\text{subject}.\text{id})$$

Two asymptomatic subjects without known TSD were excluded from the model. Association was assessed separately for females and males, and jointly by the combined contrast (Female:TSD + Male:TSD)/2. Dependent variables were converted to ranks in the model to reduce the effect of potential outliers.

Using a conservative approach, genes were classified as TSD-associated if they had an unadjusted p value < 0.05 and were excluded from subsequent analyses as specified. To determine whether the any of the baseline differential gene sets were associated with TSD (Extended Data Fig. 1a), leading edge gene (LEG) modules were derived from the union of all LEGs of the same gene set from different contrasts (see Gene set module scores). A gene set was considered stable if none of three contrasts tested in the association model were significant (using unadjusted p value threshold of 0.05).

#### Post-vaccination differential expression analysis

Similar to the workflow employed in baseline differential expression analysis, mixed-effects models were created to evaluate changes and group differences at each available timepoint after vaccination. Subjects aged 65 and above were excluded as they received a different type of vaccine. In addition to the baseline covariates, the model also accounts for the participants’ flu vaccination history within last 10 years as follows:

$${}^{\sim }0+\text{visit}:\text{group}:\text{sex+age+race+flu}.\text{vax}.\text{count}.\text{10yr+batch}.\text{effects+}(\text{1|subject}.\text{id})$$

Three types of comparisons were examined using this model:

1. **Timepoint-specific group differences**
  Similar to the contrasts in the baseline model, but for individual timepoints post vaccination (day 1 to day 100).
2. **Vaccine-induced changes in group difference**
  Similar to the timepoint-specific contrasts above, but additionally subtracting off the corresponding baseline contrast to assess changes relative to the baseline. For example, vaccine-induced changes for female COVID vs. HC differences at D1 is evaluated with the contrast: (D1.covid.Female – D1.healthy.Female) – (Baseline.covid.Female – Baseline.healthy.Female).
3. **Reversal of COVID vs. HC difference**
  Instead of using the HC subjects at the same corresponding timepoints as reference, post-vaccination samples from the COVID-recovered subjects were compared to baseline HC with the contrasts [timepoint].covid.Female – baseline.healthy.Female and [timepoint].covid.Male – baseline.healthy.Male. These contrasts can inform whether any pre-vaccination differences observed in the COVID-recovered subjects were reverted towards healthy baseline levels after vaccination. Reversal is defined as having smaller absolute effect size (using the z.std value from the dream function) at D1 and D28 after vaccination compared to the baseline absolute effect size.

P values were adjusted for multiple testing per each timepoint, assay type and contrast combination using the BH method.

#### Gene set enrichment of differentially expressed (DE) genes

Enriched gene sets were identified using the pre-ranked gene-set enrichment analysis (GSEA) algorithm implemented in the clusterProfiler R package^80^. Genes were ranked using signed −log10 p values from differential expression models. Enrichment was assessed with gene set lists from MSigDB’s Hallmark collection^81^, Blood Transcriptomic Modules^82^, and cell type gene signatures^83^. Only gene sets with 10 to 300 genes were considered. P values were adjusted per gene set list for each contrast using the BH method and gene sets with FDR < 0.05 were considered significant. Baseline enriched gene sets were derived by intersecting significant gene sets extracted from DE models using samples independently from day −7, day 0, and both days combined. Genes associated with time since diagnosis (TSD) at baseline (see Association with time since COVID-19 diagnosis; Extended Data Table 3) were excluded from the post-vaccination enrichment analyses to help segregate the effect of vaccination from natural temporal resolution of the SARS-CoV-2 infection.

#### Gene set module scores

Gene set module scores were generated from SeqRUV normalized gene expression values (see Bulk RNA-seq data processing and transformation) using gene set variation analysis (GSVA) method in GSVA R package^84^. LEG module scores representing enriched pathway activities were calculated for relevant samples using LEGs identified by GSEA to enhance signal-to-noise ratio. The average scores between days −7 and 0 were used for calculating post-vaccination changes relative to baseline.

#### Endpoint association

To evaluate the association of relevant parameters, including gene set module scores and cell frequencies, with interferon (IFN) or antibody titer fold change endpoints, the following model was applied:

$${\text{endpoint}}^{\sim }\text{group:sex + scale(parameter):group:sex + age + race + flu.vax.count.10yr }$$

The endpoint values were converted to rank to reduce the effects of potential outliers. Replicates from the same subjects were averaged.

#### Concordance in natural influenza infection cohort

A prospective cohort study with subjects profiled prior to and at least 21 days after natural influenza infection in two seasons^85^ was utilized to assess residual effects of the infection separately in males and females. Gene expression data were downloaded from GEO using the accession GSE68310. Subjects with only influenza A virus infection (n=51 females and 35 males) were identified and included for this analysis. Lowly expressed probes were removed, and the remaining data were converted to gene-based expressions. No additional processing steps were performed as the data were already normalized.

Separately for each season, differential expression analysis between baseline (pre-infection) and spring (long term post-infection) samples from the same individuals were performed using the dream function in the variancePartition R package. A mixed-effects model accounting for flu vaccination history and disease severity (based on fever grade: none, low, and high) was constructed as follows:

$${}^{\sim }0+\text{timepoint}:\text{sex+age+num}.\text{flu}.\text{vaccination+fever}.\text{grade+}(\text{1|subject}.\text{id})$$

Differentially expressed (DE) genes were identified using the contrasts Spring.F - Baseline.F and Spring.M – Baseline.M for females and males, respectively. Sex difference was evaluated by the contrast (Spring.M – Baseline.M) – (Spring.F - Baseline.F). Concordance of DE results between the two seasons were evaluated based on correlation of effect size across genes (z.std values generated by dream).

Enrichment analysis was performed to determine whether the same set of genes were differentially expressed between pre- and post-influenza infection from this independent cohort and in COVID-recovered subjects compared to healthy controls prior to vaccination. To better match the age range of subjects between the two studies, baseline differential gene analysis was performed again with subjects under 65 years of age in the COVID cohort (see Baseline differential expression analysis). Given that the males showed stronger concordance between the two flu seasons (Extended Data Fig. 2b), COVID DE genes were ranked by signed −log10 p values and tested against a gene set formed by the intersect of DE (p < 0.05) genes in males from the flu infection cohort.

### CITE-seq

#### a) Single cell CITE-seq processing

Frozen PBMC samples were thawed, recovered and washed using RPMI media with 10% FBS and 10mg/mL DNase I (STEMCELL) and then processed as previously described^86^ for CITE-seq staining. In brief, samples from different donors were pooled and different timepoints from the same donor were pooled separately so that each pool contains only one timepoint from one donor. PBMC pools were Fc blocked (Human TruStain FcX, BioLegend) and stained with Totalseq-C human ‘hashtag’ antibodies (BioLegend), washed with CITE-seq staining buffer (2% BSA in PBS). Then hashtagged PBMC pools were combined and cells were stained with a cocktail of TotalSeq-C human lyophilized panel (BioLegend) of 137 surface proteins (including 7 isotype controls, refer to repository table) and SARS-CoV-2 S1 protein probe. Then, cells were washed, resuspended in PBS, and counted before proceeding immediately to the single cell partition step.

#### b) Single cell CITE-seq library construction and sequencing

PBMC samples were partitioned into single cell Gel-Bead in Emulsion (GEM) mixed together with the reverse transcription (RT) mix using 10× 5’ Chromium Single Cell Immune Profiling Next GEM v2 chemistry (10x Genomics, Pleasanton, CA), as previously described^86^. The RT step was conducted in the Veriti^™^ Thermal Cycler (ThermoFisher Scientific, Waltham, MA). Single cell gene expression, cell surface protein, T cell receptor (TCR) and B cell receptor (BCR) libraries were prepared as instructed by 10x Genomics user guides (https://www.10xgenomics.com/resources/user-guides/). All libraries were quality controlled using Bioanalyzer (Agilent, Santa Clara, CA) and quantified using Qubit Fluorometric (ThermoFisher). 10x Genomics 5’ Single cell gene expression, cell surface protein tag, TCR and BCR libraries were pooled and sequenced on Illumina NovaSeq platform (Illumina, San Diego, CA) using the following sequencing parameters: read1–100-cycle, i7–10-, i5–10, read2–100.

### CITE-seq data processing and statistical analyses

#### a) Single cell sample demultiplexing and preprocessing

Single cell sequencing data was demultiplexed, converted to FASTQ format, mapped to human hg19 reference genome and counted using *CellRanger* (10x Genomics) pipeline. The sample level demultiplex was done based on two levels as previously described^86^: 1) Hashtag antibody staining to distinguish different timepoint samples from a same subject; 2) single nucleotide polymorphisms (SNPs) called from the whole blood RNA-seq data to identify different subjects. Specifically, *CellRanger* version 6.0.1 was used for generating count matrix and the software package *demuxlet* (v2, from the *‘popscle’* software suite)^87^ was used to match single cell gene expression data to each donor and identify empty droplets and doublets.

#### b) Single-cell data clustering and cell annotation

Single-cell data were further processed using Seurat (v4.0.3) running in R v4.1.1. We removed cells with less than 200 and greater than 5,000 detected genes, greater than 15% mitochondrial reads, cell surface protein tag greater than 60,000, and hashtag antibody counts greater than 20,000. The protein data was normalized and denoised using the DSB method^88^. The following parameters were used in the dsb normalization function: define.pseudocount = TRUE, pseudocount.use = 10, denoise_counts = TRUE, use.isotype.control = TRUE. The DSB-normalized protein data were used to generate the top variable features (n = 100) and principal components (PCs). Then the shared nearest neighbor (SNN) graph followed by k-nearest neighbors clustering were built using the FindNeighbors and FindClusters functions using first 15 PCs in Seurat (v4.0.3), respectively. Cell clusters were quality controlled based on their nearest neighbors and cell surface proteins. Cells were then further clustered within each major cell population using “weighted-nearest neighbor” (WNN) analysis in Seurat (v4.0.3) by integrating both cell surface protein and gene expression modalities. The WNN clusters were manually annotated using the surface protein together with gene expression.

#### c) Pseudobulk differential expression and gene set enrichment analysis

Pseudobulk gene differential expression analysis and gene set enrichment analysis (GSEA) were performed as described before^86^. Briefly, cells from a given sample were computationally “pooled” according to their cluster assignment by summing all reads for a given gene. Pseudobulk libraries made up by few cells and therefore likely not modeled properly by bulk differential expression methods were removed from analysis for each cell-type to remove samples that contained fewer than 4 cells and less than 35000 library size after pooling. Lowly expressed genes were removed for each cell type individually using the filterByExpr function from *edgeR*^89^. Differentially expressed genes were identified using the *limma voom* workflow which models the log of the counts per million (cpm) of each gene^76^. Scaling factors for library size normalization were calculated with the calcNormFactors function with “RLE” method. Genes were ranked using the (sign of fold change)*-log10(p-value) for the relevant coefficient from the *limma voom* model. Enriched gene sets were identified using the pre-ranked GSEA algorithm implemented in the FGSEA R package^90^.

To further digest the differences between baseline groups, the gene set list used for single-cell enrichment analysis was derived from the significance of GSEA results of whole blood RNA-seq data above (see Gene set enrichment of differentially expressed (DE) genes). The significantly enriched gene sets are from day 0 female COVID vs. healthy and day 0 male COVID vs. healthy test models (without neutrophil number correction). The Monaco gene sets were then excluded for the single-cell analysis given the cell clusters were annotated and no further cell type demultiplex needed.

##### Models used for differential expression:

Using the pseudobulk *limma voom* workflow as described above, differentially expressed genes between patient groups were identified with a model using all samples with the following formula in R: “~0 + Timepoint.sex.group + age”, where Timepoint.sex.group is a factor variable with eight levels including all timepoints (D0, D1), sex (Female, Male) and COVID-19 or healthy groups: 1) D0.Female.COVID; 2) D0.Female.healthy; 3) D0.Male.COVID; 4) D0.Male.healthy; 5) D1.Female.COVID; 6) D1.Female.healthy; 7) D1.Male.COVID; 8) D1.Male.healthy. The contrasts.fit function in *limma* was then used to compare the estimated means between different groups.

#### d) Within monocyte clustering using leading-edge genes from gene set enrichment analysis

Single monocytes were separately clustered using mRNA expression profiles of the leading-edge (LE) genes in the BTM modules “btm_M4.0_cell cycle and transcription” and “btm_M11.0_enriched in monocytes (II)” which are significantly associated with D0 COVID vs. D0 healthy in both males and females from the above GSEA analyses for the classical monocytes. Specifically, the union of the LEGs from D0.Female.COVID vs. D0.Female.healthy and D0.Male.COVID vs. D0.Male.healthy in classical monocytes were used. Clustering was performed on the scaled RNA data after regressing out subject variation using ScaleData function in *Seurat*. PCA was performed using those LEGs, and the shared nearest neighbors graph was constructed using the top 15 PCs and clustering was performed on the graph using a resolution of 1 using FindNeighbors and FindClusters functions in *Seurat*, respectively.

#### e) Single-cell module score calculation and Visualization

To visualize the differences between D0.healthy, D0.COVID and D1.COVID gene expression change in single monocytes of the BTM M4.0 and M11.0 monocyte signatures, the resolving LEGs from the two modules were used to calculate the module scores of each cell. The resolving genes were defined by genes with lower moderated T statistics for the relevant coefficient from the *limma voom* model at D1.COVID vs. D0.healthy compared to D0.COVD vs. D0.healthy. Module scores were calculated using AddModuleScore function in *Seurat*. Then the module score for each cell in a certain cluster was scaled across cells within the cluster. Cells from D0.healthy, D0.COVID and D1.COVID groups were down-sampled to the same number of cells. The UMAP embeddings of cells colored with scaled module scores were shown.

#### f) Gene set module score calculation

Module scores (gene set signature score) representing enriched pathway activities were calculated for each pseudobulk sample of certain cell types. Specifically, LEG of BTM modules M4.0 and M11.0 were identified by GSEA from 1) D0.Female.COVID vs. D0.Female.healthy and 2) D0.Male.COVID vs. D0.Male.healthy models. The genes which are also resolving at D1 (see definition above in “e) Single-cell module score calculation and Visualization”) from each model were used for the score calculation for female and male samples, respectively. The pseudobulk gene counts were normalized with the varianceStabilizingTransformation function from *DEseq2* R package^91^. The scores were then generated using gene set variation analysis (GSVA) method from the *GSVA* R package^84^.

#### g) Within monocyte clustering and single-cell module score calculation of external acute COVID-19 single-cell CITE-seq data

Single-cell data from the Brescia cohort of Liu et al.^86^ was downloaded from GEO. Single monocytes data was extracted and separately clustered using mRNA expression profiles of the same union LEGs in “d) Within monocyte clustering using leading-edge genes from gene set enrichment analysis”. The module score of each single cell was generated and visualized using those LEGs as described in “e) Single-cell module score calculation and Visualization”.

#### h) Gene set module score calculation of external acute COVID-19 single-cell CITE-seq data

Single-cell data from the Brescia cohort were pooled as described in “c) Pseudobulk differential expression and gene set enrichment analysis”. The gene set module scores of BTM modules M4.0 and M11.0 for all samples were generated using the union LEGs in “d) Within monocyte clustering using leading-edge genes from gene set enrichment analysis”. The pseudobulk gene counts were normalized with the varianceStabilizingTransformation function from *DEseq2* R package^91^. The scores were then generated using gene set variation analysis (GSVA) method from the *GSVA* R package^84^.

## Extended Data

## Supplementary Material

Supplement 1

Supplement 2

Supplement 3

Supplement 4

Supplement 5

Supplement 6

Supplement 7

Supplement 8

Supplement 9

Supplement 10

1

## Figures and Tables

**Table 1:** 

Specificity	Fluorochrome	Ab clone	vendor	cat#
H3 Probe	Alexa Fluor488			
IgM	BB700	G20–127	BD	Custom
IgA	PE	IS11–8E10	Miltenyi	130-113-476
CD21	PE-CF594	B-Ly6	BD	563474
CD85J	PE-Cy7	GHI/75	Biolegend	333712
B Probe	AlexaFluor 647			
FCRL5	R718	509F6	BD	751885
CD20	APC-Fire 750	2H7	Biolegend	302358
IgG	BUV395	G18–145	BD	564229
Dead cells	Live/Dead Blue		ThermoFisher	L23105
CD38	BUV661	HIT2	BD	612969
H1 Probe	BV421			
CD14	BV510	M5E2	Biolegend	301842
CD56	BV510	HCD56	Biolegend	318340
CD3	BV510	OKT3	Biolegend	317332
CD27	BV605	O323	Biolegend	302830
CD71	BV650	CY1G4	Biolegend	334116
CD19	BV750	SJ25C1	BD	747161
IgD	BV785	IA6–2	BD	740997

**Table 2:** 

Specificity	Fluorochrome	Ab clone	vendor	cat#
CD45RA	BUV395	5H9	BD	740315
CD16	BUV496	3G8	BD	612944
ICOS	BUV563	DX29	BD	741421
CD11c	BUV661	B-Ly6	BD	612967
CD56	BUV737	NCAM16.2	BD	612766
CD8	BUV805	SK1	BD	612889
Viability	LIVE/DEAD BLUE		Thermo	L34962
CD197 (CCR7)	BV421	G043H7	Biolegend	353208
CD123	Super Bright 436	6H6	Thermo	62-1239-42
CD161	eFluor450	HP-3G10	Thermo	48-1619-42
IgD	BV480	IA6–2	BD	566138
CD3	BV510	SK7	BioLegend	344828
CD20	Pacific Organge	HI47	Thermo	MHCD2030
IgM	BV570	MHM-88	Biolegend	314517
IgG	BV605	G18–145	BD	563246
CD28	BV650	CD28.2	Biolegend	302946
CD196 (CCR6)	BV711	G034E3	Biolegend	353436
CD185 (CXCR5)	BV750	RF8B2	BD	747111
CD279 (PD-1)	BV785	EH12.2H7	Biolegend	329929
CD141	BB515	1A4	BD	566017
CD57	FITC	HNK-1	Biolegend	359604
CD14	Spark Blue 550	63D3	Biolegend	367148
CD45	PerCP	H130	Thermo	MHCD4531
CD21	PerCP-Cy5.5	Bu32	Biolegend	354908
TCRgd	PerCP-eFluor710	B1.1	Thermo	46-9959-42
CD25	PE	BC96	Thermo	12-0259-42
CD4	CF568	SK3	Biolegend	Custom
CD24	PE/Dazzle594	ML5	Biolegend	311134
CD95 (Fas)	PE-Cy5	DX2	Thermo	15-0959-42
CD183 (CXCR3)	PE-Cy7	CEW33D	Thermo	25-1839-42
CD27	APC	O323	Thermo	17-0279-42
CD1c	Alexa Fluor 647	L161	Biolegend	331510
CD19	Spark NIR 685	HIB19	Biolegend	302270
CD127	APC-R700	HIL-7R-M21	BD	565185
HLA-DR	APC/Fire750	L243	Thermo	47-9952-42
CD38	APC/Fire810	HIT2	Biolegend	303550

## Data Availability

Raw and processed data from the whole blood bulk RNAseq are available from the NCBI Gene Expression Omnibus, accession number GEO: GSE194378 (https://www.ncbi.nlm.nih.gov/geo/query/acc.cgi?acc=GSE194378; will be released to public at the time of publication). Additional datasets, including clinical, proteomics, flow cytometry, CITE-seq, and influenza antibody measurements, are available at: https://doi.org/10.5281/zenodo.5935845 (will be released to public at time of publication). The influenza infection dataset we utilized was downloaded directly from GEO: GSE68310 (https://www.ncbi.nlm.nih.gov/geo/query/acc.cgi?acc=GSE68310).

## References

[1] Nalbandian A. Post-acute COVID-19 syndrome. Nat. Med. 27, 601–615 (2021). 33753937 10.1038/s41591-021-01283-z PMC8893149

[2] Mina M. J. Measles virus infection diminishes preexisting antibodies that offer protection from other pathogens. Science 366, 599–606 (2019). 31672891 10.1126/science.aay6485 PMC8590458

[3] Petrova V. N. Incomplete genetic reconstitution of B cell pools contributes to prolonged immunosuppression after measles. Sci. Immunol. 4, eaay6125 (2019). 31672862 10.1126/sciimmunol.aay6125

[4] Kalish H. Undiagnosed SARS-CoV-2 seropositivity during the first 6 months of the COVID-19 pandemic in the United States. Sci. Transl. Med. 13, eabh3826 (2021). 34158410 10.1126/scitranslmed.abh3826 PMC8432952

[5] WHO Coronavirus (COVID-19) Dashboard. https://covid19.who.int/.

[6] Sudre C. H. Attributes and predictors of long COVID. Nat. Med. 27, 626–631 (2021). 33692530 10.1038/s41591-021-01292-y PMC7611399

[7] Wheatley A. K. Evolution of immune responses to SARS-CoV-2 in mild-moderate COVID-19. Nat. Commun. 12, 1162 (2021). 33608522 10.1038/s41467-021-21444-5 PMC7896046

[8] Patel H. H., Patel H. R. & Higgins J. M. Modulation of red blood cell population dynamics is a fundamental homeostatic response to disease. Am. J. Hematol. 90, 422–428 (2015). 25691355 10.1002/ajh.23982 PMC4717489

[9] Salvagno G. L., Sanchis-Gomar F., Picanza A. & Lippi G. Red blood cell distribution width: A simple parameter with multiple clinical applications. Crit. Rev. Clin. Lab. Sci. 52, 86–105 (2015). 25535770 10.3109/10408363.2014.992064

[10] Kubánková M. Physical phenotype of blood cells is altered in COVID-19. Biophys. J. 120, 2838–2847 (2021). 34087216 10.1016/j.bpj.2021.05.025 PMC8169220

[11] Cai Y. Kynurenic acid may underlie sex-specific immune responses to COVID-19. Sci. Signal. 14, eabf8483 (2021). 34230210 10.1126/scisignal.abf8483 PMC8432948

[12] Gallais F. Evolution of antibody responses up to 13 months after SARS-CoV-2 infection and risk of reinfection. EBioMedicine 71, 103561 (2021). 34455390 10.1016/j.ebiom.2021.103561 PMC8390300

[13] Grzelak L. Sex Differences in the Evolution of Neutralizing Antibodies to Severe Acute Respiratory Syndrome Coronavirus 2. J. Infect. Dis. 224, 983–988 (2021). 33693749 10.1093/infdis/jiab127 PMC7989436

[14] Hou Y. Multimodal single-cell omics analysis identifies epithelium-immune cell interactions and immune vulnerability associated with sex differences in COVID-19. Signal Transduct. Target. Ther. 6, 292 (2021). 34330889 10.1038/s41392-021-00709-x PMC8322111

[15] Mahallawi W. H., Alsamiri A. D., Dabbour A. F., Alsaeedi H. & Al-Zalabani A. H. Association of Viral Load in SARS-CoV-2 Patients With Age and Gender. Front. Med. 8, 39 (2021). 10.3389/fmed.2021.608215 PMC7873591 33585523

[16] Stephenson E. Single-cell multi-omics analysis of the immune response in COVID-19. Nat. Med. 27, 904–916 (2021). 33879890 10.1038/s41591-021-01329-2 PMC8121667

[17] Takahashi T. & Iwasaki A. Sex differences in immune responses. Science 371, 347–348 (2021). 33479140 10.1126/science.abe7199

[18] Ursin R. L. & Klein S. L. Sex Differences in Respiratory Viral Pathogenesis and Treatments. Annu. Rev. Virol. 8, 393–414 (2021). 34081540 10.1146/annurev-virology-091919-092720

[19] Yu C. Mucosal-associated invariant T cell responses differ by sex in COVID-19. Med 2, 755–772.e5 (2021). 33870241 10.1016/j.medj.2021.04.008 PMC8043578

[20] Zhai Y. Host Transcriptional Response to Influenza and Other Acute Respiratory Viral Infections – A Prospective Cohort Study. PLOS Pathog. 11, e1004869 (2015). 26070066 10.1371/journal.ppat.1004869 PMC4466531

[21] Pérez-Gómez A. Dendritic cell deficiencies persist seven months after SARS-CoV-2 infection. Cell. Mol. Immunol. 18, 2128–2139 (2021). 34290398 10.1038/s41423-021-00728-2 PMC8294321

[22] Laing A. G. A dynamic COVID-19 immune signature includes associations with poor prognosis. Nat. Med. 26, 1623–1635 (2020). 32807934 10.1038/s41591-020-1038-6

[23] Liu C. Time-resolved systems immunology reveals a late juncture linked to fatal COVID-19. Cell 184, 1836–1857.e22 (2021). 33713619 10.1016/j.cell.2021.02.018 PMC7874909

[24] Nakaya H. I. Systems Analysis of Immunity to Influenza Vaccination across Multiple Years and in Diverse Populations Reveals Shared Molecular Signatures. Immunity 43, 1186–1198 (2015). 26682988 10.1016/j.immuni.2015.11.012 PMC4859820

[25] Pulendran B. Systems vaccinology: Probing humanity’s diverse immune systems with vaccines. Proc. Natl. Acad. Sci. 111, 12300–12306 (2014). 25136102 10.1073/pnas.1400476111 PMC4151766

[26] Tsang J. S. Utilizing population variation, vaccination, and systems biology to study human immunology. Trends Immunol. 36, 479–493 (2015). 26187853 10.1016/j.it.2015.06.005 PMC4979540

[27] Tsang J. S. Global Analyses of Human Immune Variation Reveal Baseline Predictors of Postvaccination Responses. Cell 157, 499–513 (2014). 24725414 10.1016/j.cell.2014.03.031 PMC4139290

[28] Moa A. M., Chughtai A. A., Muscatello D. J., Turner R. M. & MacIntyre C. R. Immunogenicity and safety of inactivated quadrivalent influenza vaccine in adults: A systematic review and meta-analysis of randomised controlled trials. Vaccine 34, 4092–4102 (2016). 27381642 10.1016/j.vaccine.2016.06.064

[29] Ravichandran S. Longitudinal antibody repertoire in ‘mild’ versus ‘severe’ COVID-19 patients reveals immune markers associated with disease severity and resolution. Sci. Adv. 7, eabf2467 (2021). 33674317 10.1126/sciadv.abf2467 PMC7935365

[30] Ravichandran S. SARS-CoV-2 immune repertoire in MIS-C and pediatric COVID-19. Nat. Immunol. 22, 1452–1464 (2021). 34611361 10.1038/s41590-021-01051-8

[31] Ellebedy A. H. Defining antigen-specific plasmablast and memory B cell subsets in blood following viral infection and vaccination of humans. Nat. Immunol. 17, 1226–1234 (2016). 27525369 10.1038/ni.3533 PMC5054979

[32] Arneborn P., Biberfeld G., Forsgren M. & von Stedingk L. V. Specific and non-specific B cell activation in measles and varicella. Clin. Exp. Immunol. 51, 165–172 (1983). 6299636 PMC1536744

[33] Horns F., Dekker C. L. & Quake S. R. Memory B Cell Activation, Broad Anti-influenza Antibodies, and Bystander Activation Revealed by Single-Cell Transcriptomics. Cell Rep. 30, 905–913.e6 (2020). 31968262 10.1016/j.celrep.2019.12.063 PMC7891556

[34] Valvezan A. J. & Manning B. D. Molecular logic of mTORC1 signalling as a metabolic rheostat. Nat. Metab. 1, 321–333 (2019). 32694720 10.1038/s42255-019-0038-7 PMC12569966

[35] Goodridge H. S. Harnessing the beneficial heterologous effects of vaccination. Nat. Rev. Immunol. 16, 392–400 (2016). 27157064 10.1038/nri.2016.43 PMC4931283

[36] Netea M. G. Defining trained immunity and its role in health and disease. Nat. Rev. Immunol. 20, 375–388 (2020). 32132681 10.1038/s41577-020-0285-6 PMC7186935

[37] Wimmers F. The single-cell epigenomic and transcriptional landscape of immunity to influenza vaccination. Cell 184, 3915–3935.e21 (2021). 34174187 10.1016/j.cell.2021.05.039 PMC8316438

[38] Stoeckius M. Simultaneous epitope and transcriptome measurement in single cells. Nat. Methods 14, 865–868 (2017). 28759029 10.1038/nmeth.4380 PMC5669064

[39] Li S. Molecular signatures of antibody responses derived from a systems biology study of five human vaccines. Nat. Immunol. 15, 195–204 (2014). 24336226 10.1038/ni.2789 PMC3946932

[40] Arunachalam P. S. Systems biological assessment of immunity to mild versus severe COVID-19 infection in humans. Science 369, 1210–1220 (2020). 32788292 10.1126/science.abc6261 PMC7665312

[41] Paludan S. R. & Mogensen T. H. Innate immunological pathways in COVID-19 pathogenesis. Sci. Immunol. (2022) doi:10.1126/sciimmunol.abm5505. 34995097

[42] Reyes M. Plasma from patients with bacterial sepsis or severe COVID-19 induces suppressive myeloid cell production from hematopoietic progenitors in vitro. Sci. Transl. Med. (2021) doi:10.1126/scitranslmed.abe9599. PMC8432955 34103408

[43] Schulte-Schrepping J. Severe COVID-19 Is Marked by a Dysregulated Myeloid Cell Compartment. Cell 182, 1419–1440.e23 (2020). 32810438 10.1016/j.cell.2020.08.001 PMC7405822

[44] Schultze J. L. & Aschenbrenner A. C. COVID-19 and the human innate immune system. Cell 184, 1671–1692 (2021). 33743212 10.1016/j.cell.2021.02.029 PMC7885626

[45] Phetsouphanh C. Immunological dysfunction persists for 8 months following initial mild-to-moderate SARS-CoV-2 infection. Nat. Immunol. 1–7 (2022) doi:10.1038/s41590-021-01113-x. 35027728

[46] Sette A. & Crotty S. Adaptive immunity to SARS-CoV-2 and COVID-19. Cell 184, 861–880 (2021). 33497610 10.1016/j.cell.2021.01.007 PMC7803150

[47] Utrero-Rico A. Alterations in Circulating Monocytes Predict COVID-19 Severity and Include Chromatin Modifications Still Detectable Six Months after Recovery. Biomedicines 9, 1253 (2021). 34572439 10.3390/biomedicines9091253 PMC8471575

[48] Wong L.-Y. R. & Perlman S. Immune dysregulation and immunopathology induced by SARS-CoV-2 and related coronaviruses - are we our own worst enemy? Nat. Rev. Immunol. (2021) doi:10.1038/s41577-021-00656-2. PMC8617551 34837062

[49] You M. Single-cell epigenomic landscape of peripheral immune cells reveals establishment of trained immunity in individuals convalescing from COVID-19. Nat. Cell Biol. 23, 620–630 (2021). 34108657 10.1038/s41556-021-00690-1 PMC9105401

[50] Huang C. Clinical features of patients infected with 2019 novel coronavirus in Wuhan, China. The Lancet 395, 497–506 (2020). 10.1016/S0140-6736(20)30183-5 PMC7159299 31986264

[51] Hoehn K. B. Cutting Edge: Distinct B Cell Repertoires Characterize Patients with Mild and Severe COVID-19. J. Immunol. Baltim. Md 1950 206, 2785–2790 (2021). 10.4049/jimmunol.2100135 PMC8627528 34049971

[52] Huang L. Dynamic blood single-cell immune responses in patients with COVID-19. Signal Transduct. Target. Ther. 6, 110 (2021). 33677468 10.1038/s41392-021-00526-2 PMC7936231

[53] Talla A. Longitudinal immune dynamics of mild COVID-19 define signatures of recovery and persistence. BioRxiv Prepr. Serv. Biol. 2021.05.26.442666 (2021) doi:10.1101/2021.05.26.442666.

[54] Takahashi T. Sex differences in immune responses that underlie COVID-19 disease outcomes. Nature 588, 315–320 (2020). 32846427 10.1038/s41586-020-2700-3 PMC7725931

[55] Klein S. L. & Flanagan K. L. Sex differences in immune responses. Nat. Rev. Immunol. 16, 626–638 (2016). 27546235 10.1038/nri.2016.90

[56] Su Y. Multiple Early Factors Anticipate Post-Acute COVID-19 Sequelae. Cell 0, (2022). 10.1016/j.cell.2022.01.014 PMC8786632 35216672

[57] Habibi M. S. Neutrophilic inflammation in the respiratory mucosa predisposes to RSV infection. Science 370, eaba9301 (2020). 33033192 10.1126/science.aba9301 PMC7613218

[58] Messina N. L., Zimmermann P. & Curtis N. The impact of vaccines on heterologous adaptive immunity. Clin. Microbiol. Infect. 25, 1484–1493 (2019). 30797062 10.1016/j.cmi.2019.02.016

[59] Arunachalam P. S. Systems vaccinology of the BNT162b2 mRNA vaccine in humans. Nature 596, 410–416 (2021). 34252919 10.1038/s41586-021-03791-x PMC8761119

[60] De Mot L. D. Transcriptional profiles of adjuvanted hepatitis B vaccines display variable interindividual homogeneity but a shared core signature. Sci. Transl. Med. (2020) doi:10.1126/scitranslmed.aay8618. 33177181

[61] Brauns E. Functional reprogramming of monocytes in acute and convalescent severe COVID-19 patients. available at Research Square 10.21203/rs.3.rs-766032/v1. (2021). PMC9090263 35380990

[62] Kotliarov Y. Broad immune activation underlies shared set point signatures for vaccine responsiveness in healthy individuals and disease activity in patients with lupus. Nat. Med. 26, 618–629 (2020). 32094927 10.1038/s41591-020-0769-8 PMC8392163

[63] Harris P. A. Research electronic data capture (REDCap)—A metadata-driven methodology and workflow process for providing translational research informatics support. J. Biomed. Inform. 42, 377–381 (2009). 18929686 10.1016/j.jbi.2008.08.010 PMC2700030

[64] Harris P. A. The REDCap consortium: Building an international community of software platform partners. J. Biomed. Inform. 95, 103208 (2019). 31078660 10.1016/j.jbi.2019.103208 PMC7254481

[65] Ravichandran S. Antibody signature induced by SARS-CoV-2 spike protein immunogens in rabbits. Sci. Transl. Med. 12, eabc3539 (2020). 32513867 10.1126/scitranslmed.abc3539 PMC7286538

[66] Ravichandran S. Longitudinal antibody repertoire in ‘mild’ versus ‘severe’ COVID-19 patients reveals immune markers associated with disease severity and resolution. Sci. Adv. 7, eabf2467 (2021). 33674317 10.1126/sciadv.abf2467 PMC7935365

[67] Tang J. Antibody affinity maturation and plasma IgA associate with clinical outcome in hospitalized COVID-19 patients. Nat. Commun. 12, 1221 (2021). 33619281 10.1038/s41467-021-21463-2 PMC7900119

[68] Khurana S. MF59 adjuvant enhances diversity and affinity of antibody-mediated immune response to pandemic influenza vaccines. Sci. Transl. Med. 3, 85ra48 (2011). 10.1126/scitranslmed.3002336 PMC3501657 21632986

[69] Khurana S. Human antibody repertoire after VSV-Ebola vaccination identifies novel targets and virus-neutralizing IgM antibodies. Nat. Med. 22, 1439–1447 (2016). 27798615 10.1038/nm.4201

[70] Khurana S. Repeat vaccination reduces antibody affinity maturation across different influenza vaccine platforms in humans. Nat. Commun. 10, 3338 (2019). 31350391 10.1038/s41467-019-11296-5 PMC6659679

[71] De Biasi S. Marked T cell activation, senescence, exhaustion and skewing towards TH17 in patients with COVID-19 pneumonia. Nat. Commun. 11, 3434 (2020). 32632085 10.1038/s41467-020-17292-4 PMC7338513

[72] Heit A. Vaccination establishes clonal relatives of germinal center T cells in the blood of humans. J. Exp. Med. 214, 2139–2152 (2017). 28637884 10.1084/jem.20161794 PMC5502430

[73] Park L. M., Lannigan J. & Jaimes M. C. OMIP-069: Forty-Color Full Spectrum Flow Cytometry Panel for Deep Immunophenotyping of Major Cell Subsets in Human Peripheral Blood. Cytom. Part J. Int. Soc. Anal. Cytol. 97, 1044–1051 (2020). 10.1002/cyto.a.24213 PMC8132182 32830910

[74] Bolger A. M., Lohse M. & Usadel B. Trimmomatic: a flexible trimmer for Illumina sequence data. Bioinforma. Oxf. Engl. 30, 2114–2120 (2014). 10.1093/bioinformatics/btu170 PMC4103590 24695404

[75] Liao Y., Smyth G. K. & Shi W. featureCounts: an efficient general purpose program for assigning sequence reads to genomic features. Bioinforma. Oxf. Engl. 30, 923–930 (2014). 10.1093/bioinformatics/btt656 24227677

[76] Law C. W., Chen Y., Shi W. & Smyth G. K. voom: Precision weights unlock linear model analysis tools for RNA-seq read counts. Genome Biol. 15, R29 (2014). 24485249 10.1186/gb-2014-15-2-r29 PMC4053721

[77] Risso D., Ngai J., Speed T. P. & Dudoit S. Normalization of RNA-seq data using factor analysis of control genes or samples. Nat. Biotechnol. 32, 896–902 (2014). 25150836 10.1038/nbt.2931 PMC4404308

[78] Tsang J. S. Global Analyses of Human Immune Variation Reveal Baseline Predictors of Postvaccination Responses. Cell 157, 499–513 (2014). 24725414 10.1016/j.cell.2014.03.031 PMC4139290

[79] Hoffman G. E. & Roussos P. Dream: powerful differential expression analysis for repeated measures designs. Bioinforma. Oxf. Engl. 37, 192–201 (2021). 10.1093/bioinformatics/btaa687 PMC8055218 32730587

[80] Wu T. clusterProfiler 4.0: A universal enrichment tool for interpreting omics data. Innov. N. Y. N 2, 100141 (2021). 10.1016/j.xinn.2021.100141 PMC8454663 34557778

[81] Liberzon A. Molecular signatures database (MSigDB) 3.0. Bioinformatics 27, 1739–1740 (2011). 21546393 10.1093/bioinformatics/btr260 PMC3106198

[82] Li S. Molecular signatures of antibody responses derived from a systems biology study of five human vaccines. Nat. Immunol. 15, 195–204 (2014). 24336226 10.1038/ni.2789 PMC3946932

[83] Monaco G. RNA-Seq Signatures Normalized by mRNA Abundance Allow Absolute Deconvolution of Human Immune Cell Types. Cell Rep. 26, 1627–1640.e7 (2019). 30726743 10.1016/j.celrep.2019.01.041 PMC6367568

[84] Hänzelmann S., Castelo R. & Guinney J. GSVA: gene set variation analysis for microarray and RNA-seq data. BMC Bioinformatics 14, 7 (2013). 23323831 10.1186/1471-2105-14-7 PMC3618321

[85] Zhai Y. Host Transcriptional Response to Influenza and Other Acute Respiratory Viral Infections – A Prospective Cohort Study. PLOS Pathog. 11, e1004869 (2015). 26070066 10.1371/journal.ppat.1004869 PMC4466531

[86] Liu C. Time-resolved systems immunology reveals a late juncture linked to fatal COVID-19. Cell 184, 1836–1857.e22 (2021). 33713619 10.1016/j.cell.2021.02.018 PMC7874909

[87] Kang H. M. Multiplexed droplet single-cell RNA-sequencing using natural genetic variation. Nat. Biotechnol. 36, 89–94 (2018). 29227470 10.1038/nbt.4042 PMC5784859

[88] Mule M. P., Martins A. J. & Tsang J. S. Normalizing and denoising protein expression data from droplet-based single cell profiling. bioRxiv (2021) doi:10.1101/2020.02.24.963603. PMC9018908 35440536

[89] McCarthy D. J., Chen Y. & Smyth G. K. Differential expression analysis of multifactor RNA-Seq experiments with respect to biological variation. Nucleic Acids Res. 40, 4288–4297 (2012). 22287627 10.1093/nar/gks042 PMC3378882

[90] Korotkevich G. Fast gene set enrichment analysis. bioRxiv (2021).

[91] Love M. I., Huber W. & Anders S. Moderated estimation of fold change and dispersion for RNA-seq data with DESeq2. Genome Biol. 15, 550 (2014). 25516281 10.1186/s13059-014-0550-8 PMC4302049

