# Supplementary material for: Influenza vaccination and single cell multiomics reveal sex dimorphic immune imprints of prior mild COVID-19
Source: medRxiv. 2022 Sep 1:2022.02.17.22271138. Originally published 2022 Feb 22. Preprint. [Version 2] doi: 10.1101/2022.02.17.22271138 (PMC8887138; doi:10.1101/2022.02.17.22271138)
Supplement: Extended Data Table 1 [file extended_data_table_1.pdf]

Extended Data Table 1. Cohort Characteristics

	COVR		Healthy	
	**Female**	**Male**	**Female**	**Male**
Subject count	17	16	21	19
**Age (Years)**				
Median	40.2	43.7	52.5	47.6
Mean	44.9	43.7	47.2	47.4
Min	23.4	21.9	22.5	24.0
Max	70.5	67.3	70.4	69.1
Aged > 65	2	2	5	5
**Race**				
Asian	1	0	2	2
Black	1	0	4	0
Multiple race	0	2	1	3
White	15	14	14	14
**Number of Influenza Vaccination in Past 10 Years**				
0	0 (0%)	1 (6.25%)	0 (0%)	2 (10.53%)
1	1 (5.88%)	0 (0%)	1 (4.76%)	0 (0%)
2	0 (0%)	1 (6.25%)	2 (9.52%)	2 (10.53%)
3	1 (5.88%)	0 (0%)	1 (4.76%)	0 (0%)
4	0 (0%)	0 (0%)	2 (9.52%)	0 (0%)
5	3 (17.65%)	3 (18.75%)	1 (4.76%)	0 (0%)
6	1 (5.88%)	1 (6.25%)	1 (4.76%)	0 (0%)
7	3 (17.65%)	0 (0%)	1 (4.76%)	1 (5.26%)
8	0 (0%)	1 (6.25%)	1 (4.76%)	3 (15.79%)
9	1 (5.88%)	1 (6.25%)	1 (4.76%)	1 (5.26%)
10	7 (41.18%)	8 (50%)	10 (47.62%)	10 (52.63%)
Experienced side effects after vaccination	16 (94.1%)	9 (56.3%)	17 (90.0%)	15 (78.9%)
**COVID-19 Symptoms**				
Asymptomatic	1 (5.9%)	1 (6.3%)	-	-
Symptomatic	16 (94.1%)	15 (93.8%)		
**Time since COVID-19 Diagnosis (Days) ***				
Median	172.0	186.0	-	-
Mean	152.9	149.3	-	-
Min	58.0	44.0	-	-
Max	237.0	248.0	-	-
**Duration of acute COVID-19 symptoms (Days) ***				
Median	14	10		
Mean	19.62	13.07		
Min	4	1		
Max	87	33		

Experienced COVID-19 residual symptoms at time of screening	8 (47.1%)	3 (18.8%)	-	-
**COVID-19 Residual Symptoms**				
Brain fog	1 (5.9%)	0 (0%)	-	-
Fatigue	2 (11.8%)	0 (0%)	-	-
Loose stools	0 (0%)	1 (6.3%)	-	-
Reduced sense of taste	1 (5.9%)	1 (6.3%)	-	-
Reduced sense of smell / smell disturbances	5 (29.4%)	2 (12.5%)	-	-
Shortness of breath / Chest pressure	1 (5.9%)	0 (0%)	-	-

* Excluding asymptomatic subjects
