# Supplementary material for: Influenza vaccination and single cell multiomics reveal sex dimorphic immune imprints of prior mild COVID-19
Source: medRxiv. 2022 Sep 1:2022.02.17.22271138. Originally published 2022 Feb 22. Preprint. [Version 2] doi: 10.1101/2022.02.17.22271138 (PMC8887138; doi:10.1101/2022.02.17.22271138)
Supplement: 1 [file NIHPP2022.02.17.22271138V2-supplement-1.pdf]

medRxiv preprint doi: https://doi.org/10.1101/2022.02.17.22271138; this version posted September 1, 2022. The copyright holder for this preprint (which was not certified by peer review) is the author/funder, who has granted medRxiv a license to display the preprint in perpetuity.
This article is a US Government work. It is not subject to copyright under 17 USC 105 and is also made available for use under a CC0 license.

Supplementary Information Fig. 1

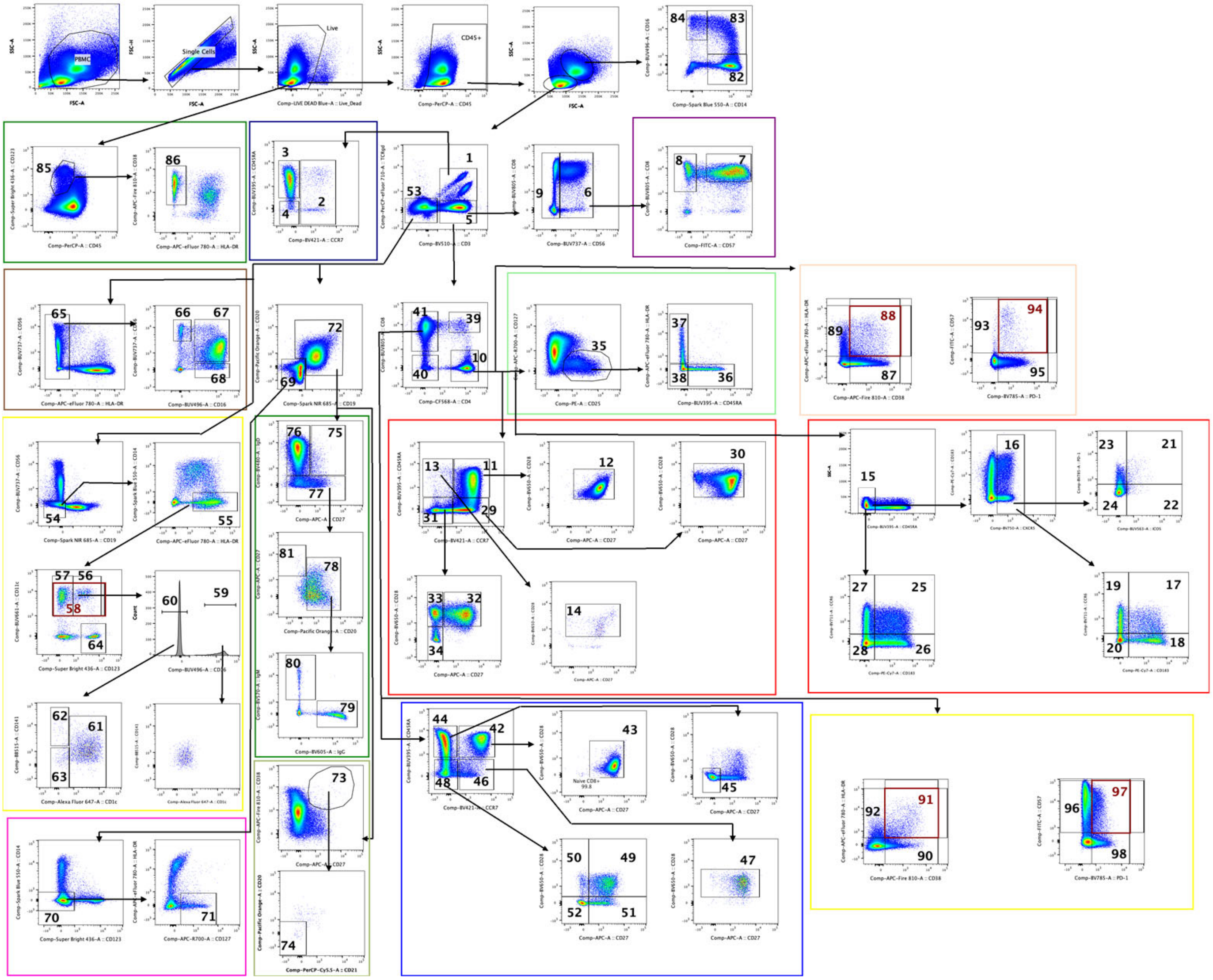

medRxiv preprint doi: https://doi.org/10.1101/2022.02.17.22271138; this version posted September 1, 2022. The copyright holder for this preprint (which was not certified by peer review) is the author/funder, who has granted medRxiv a license to display the preprint in perpetuity.
This article is a US Government work. It is not subject to copyright under 17 USC 105 and is also made available for use under a CC0 license.

Supplementary Information Figure 1: Gating strategy for the Cytek 36-color panel run on peripheral blood mononuclear cells.

medRxiv preprint doi: https://doi.org/10.1101/2022.02.17.22271138; this version posted September 1, 2022. The copyright holder for this preprint (which was not certified by peer review) is the author/funder, who has granted medRxiv a license to display the preprint in perpetuity.
This article is a US Government work. It is not subject to copyright under 17 USC 105 and is also made available for use under a CC0 license.

Supplementary Information Fig. 2

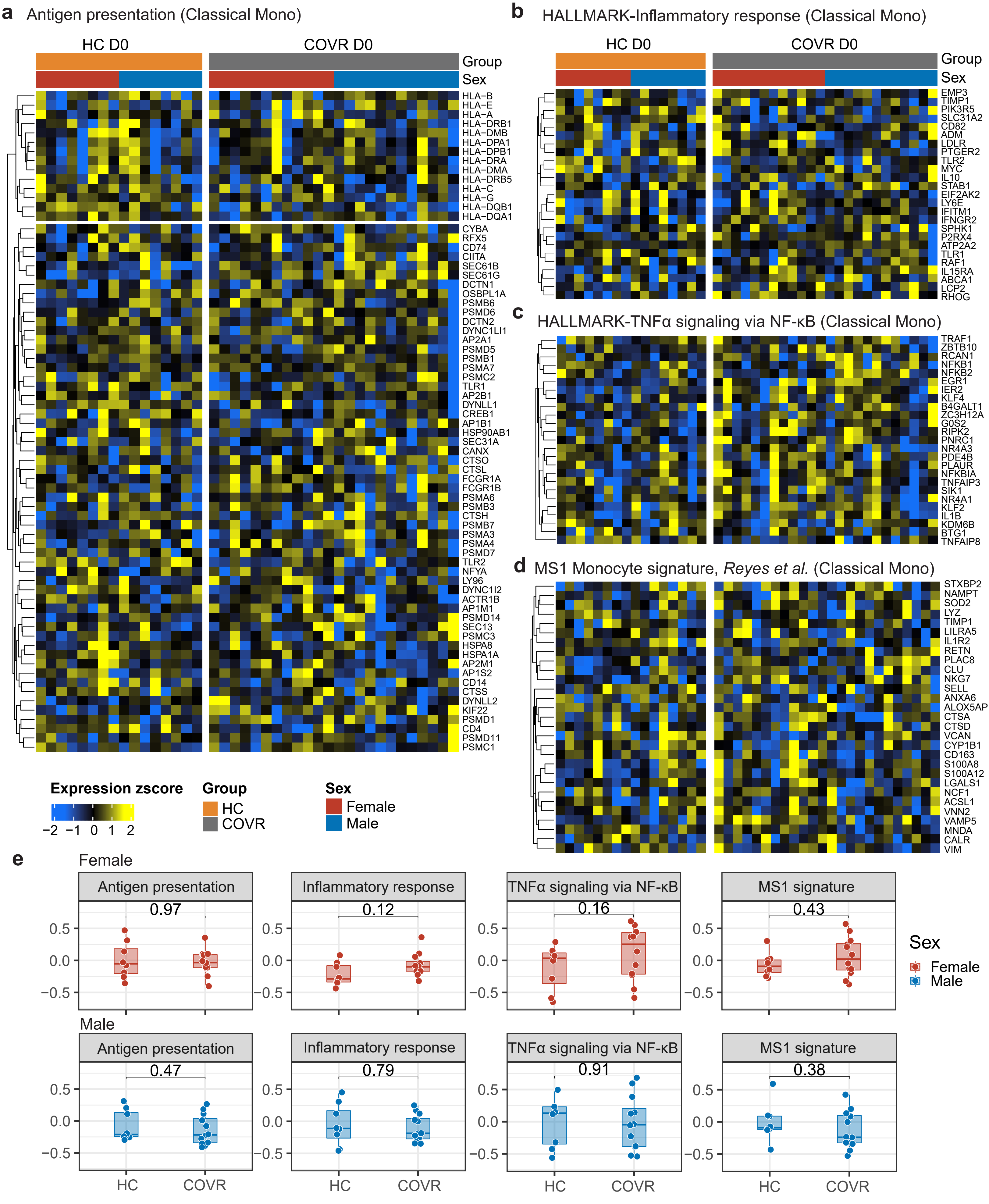

medRxiv preprint doi: https://doi.org/10.1101/2022.02.17.22271138; this version posted September 1, 2022. The copyright holder for this preprint (which was not certified by peer review) is the author/funder, who has granted medRxiv a license to display the preprint in perpetuity. This article is a US Government work. It is not subject to copyright under 17 USC 105 and is also made available for use under a CC0 license.

Supplementary Information Figure 2. Gene expression profile of antigen presentation, NF-κB/inflammatory, and monocytic myeloid-derived suppressor cell (MDSC) related signatures in classical monocytes

a, Heatmap showing the pseudobulk expression of the leading-edge genes (LEGs) from antigen presentation related gene sets, separately for male (M) and female (F), in classical monocytes from the CITE-seq day 0 (D0) pseudobulk data. The LEGs are from the acute COVID-19 vs. healthy control (HC) GSEA analysis in Liu *et al*[19], which showed that genes in the antigen presentation gene sets – KEGG Antigen processing and presentation, Reactome Antigen processing-Cross presentation, and Reactome MHC class II antigen presentation – tend to be lower in COVID-19. Samples (columns) are grouped by sex and subject group [HC at D0 and COVID-19-recovered (COVR) at D0 as indicated by the bars above the heatmap]. Gene names are shown on the right.

b, **S**imilar to **(a)**, but showing the LEGs of the "Hallmark Inflammatory response" gene set.

c, Similar to **(a)**, but showing the LEGs of the "Hallmark TNFα signaling via NF-κB" gene set derived from the acute COVID-19 vs. HC GSEA analysis in Liu *et al*[19].

d, Similar to **(a)**, but showing the genes of MSDC/MS1 monocyte signature from Reyes *et al*[22].

e, Box plots showing the module scores of the LEGs of the gene sets in (**a-d**) separately for F (top row) and M (bottom row) for the indicated subject groups (columns), in classical monocytes from the CITE-seq D0 pseudobulk data. Each dot represents a sample. P values shown are from two-tailed Wilcoxon tests of the indicated two group comparisons.

medRxiv preprint doi: https://doi.org/10.1101/2022.02.17.22271138; this version posted September 1, 2022. The copyright holder for this preprint (which was not certified by peer review) is the author/funder, who has granted medRxiv a license to display the preprint in perpetuity.
This article is a US Government work. It is not subject to copyright under 17 USC 105 and is also made available for use under a CC0 license.

Supplementary Information Fig. 3

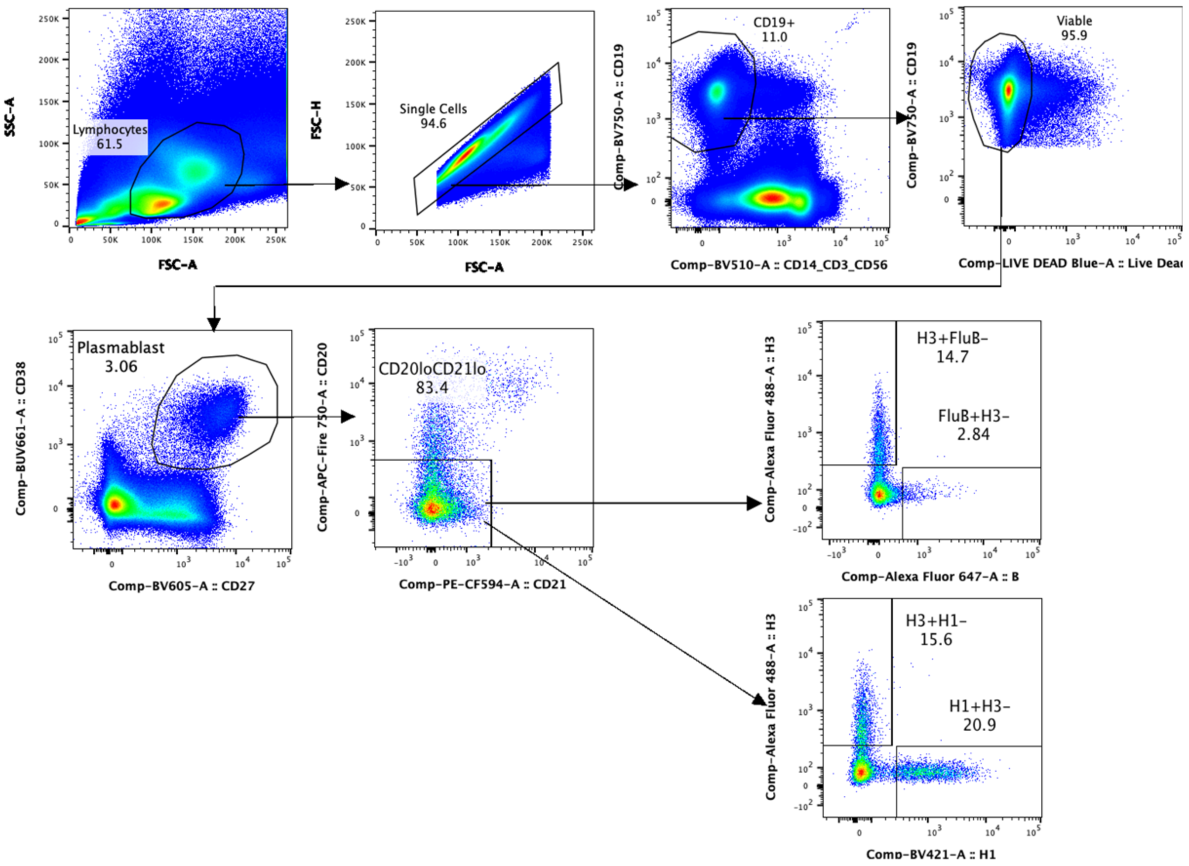

medRxiv preprint doi: https://doi.org/10.1101/2022.02.17.22271138; this version posted September 1, 2022. The copyright holder for this preprint
(which was not certified by peer review) is the author/funder, who has granted medRxiv a license to display the preprint in perpetuity.
This article is a US Government work. It is not subject to copyright under 17 USC 105 and is also made available
for use under a CC0 license.

Supplementary Information Figure 3: Gating strategies for the influenza-specific plasmablast populations.
