# Supplementary material for: Influenza vaccination reveals and partly reverses sex dimorphic immune imprints associated with prior mild COVID-19
Source: medRxiv. 2022 Feb 22:2022.02.17.22271138. Preprint. [Version 1] doi: 10.1101/2022.02.17.22271138 (PMC8887138; doi:10.1101/2022.02.17.22271138)
Supplement: Supplementary file 1 [file NIHPP2022.02.17.22271138v1-supplement-1.pdf]

medRxiv preprint doi: https://doi.org/10.1101/2022.02.17.22271138; this version posted February 22, 2022. The copyright holder for this preprint (which was not certified by peer review) is the author/funder, who has granted medRxiv a license to display the preprint in perpetuity.
This article is a US Government work. It is not subject to copyright under 17 USC 105 and is also made available for use under a CC0 license.

Supplementary Information Fig. 1

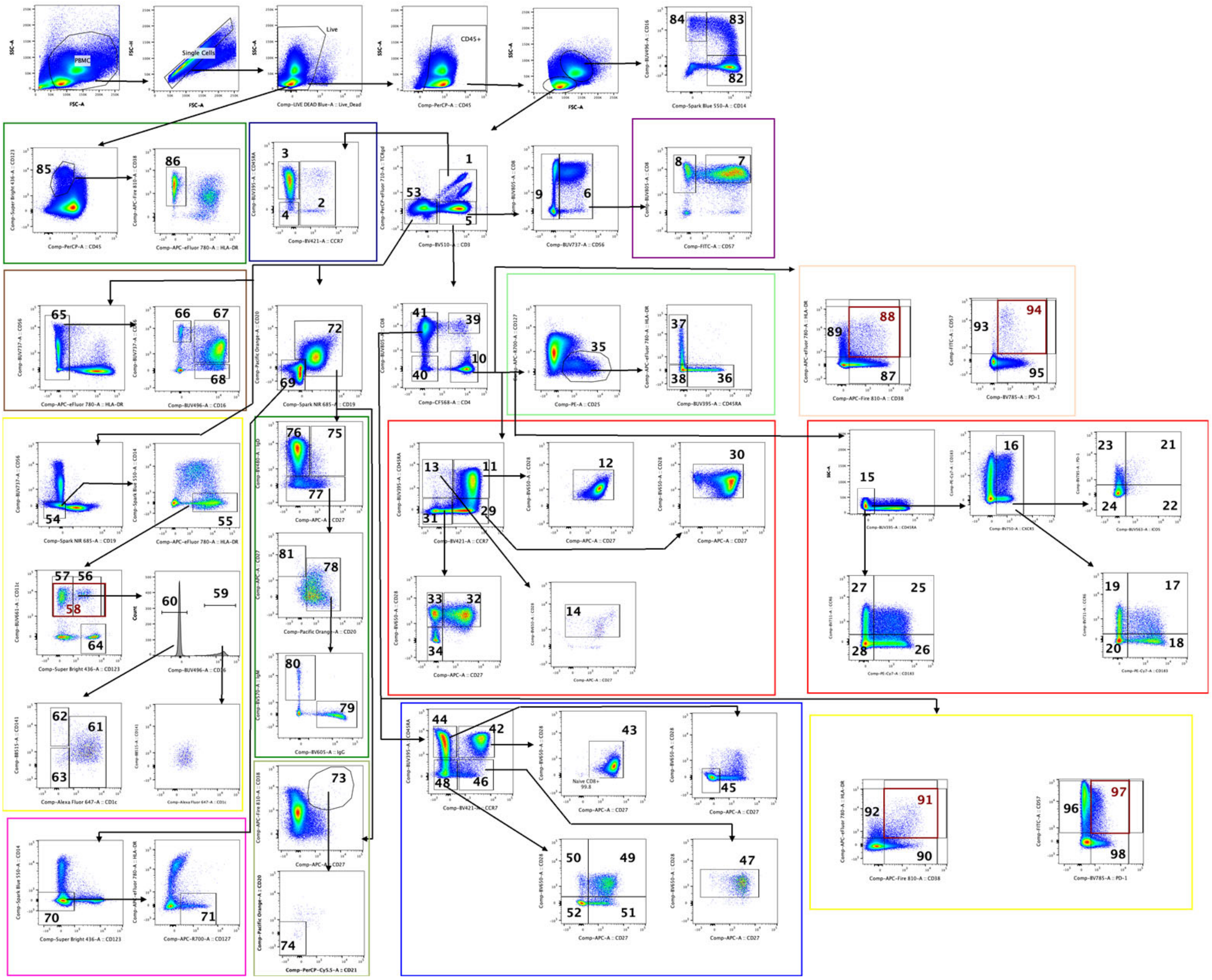

medRxiv preprint doi: https://doi.org/10.1101/2022.02.17.22271138; this version posted February 22, 2022. The copyright holder for this preprint (which was not certified by peer review) is the author/funder, who has granted medRxiv a license to display the preprint in perpetuity.
This article is a US Government work. It is not subject to copyright under 17 USC 105 and is also made available for use under a CC0 license.

Supplementary Information Figure 1: Gating strategy for the Cytek 36-color panel.

medRxiv preprint doi: https://doi.org/10.1101/2022.02.17.22271138; this version posted February 22, 2022. The copyright holder for this preprint (which was not certified by peer review) is the author/funder, who has granted medRxiv a license to display the preprint in perpetuity.
This article is a US Government work. It is not subject to copyright under 17 USC 105 and is also made available for use under a CC0 license.

Supplementary Information Fig. 2

a

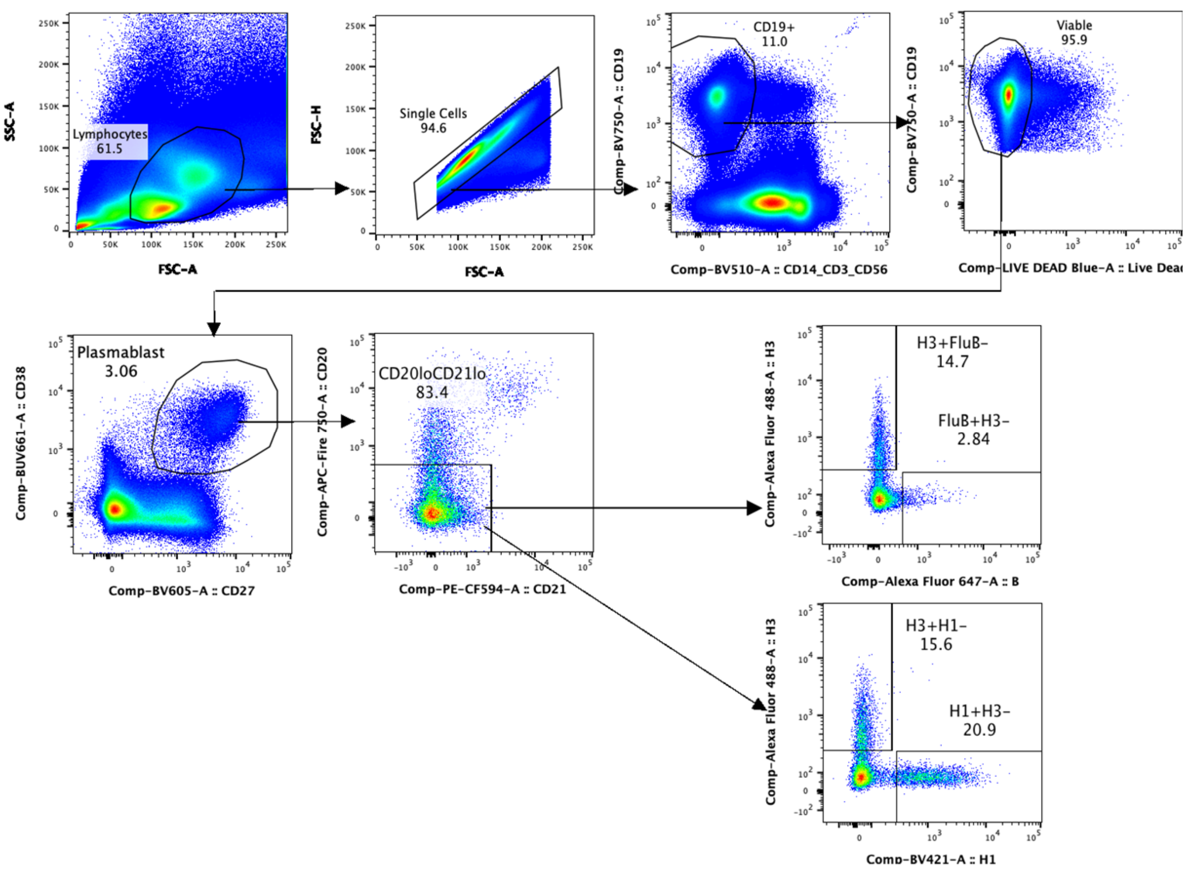

b

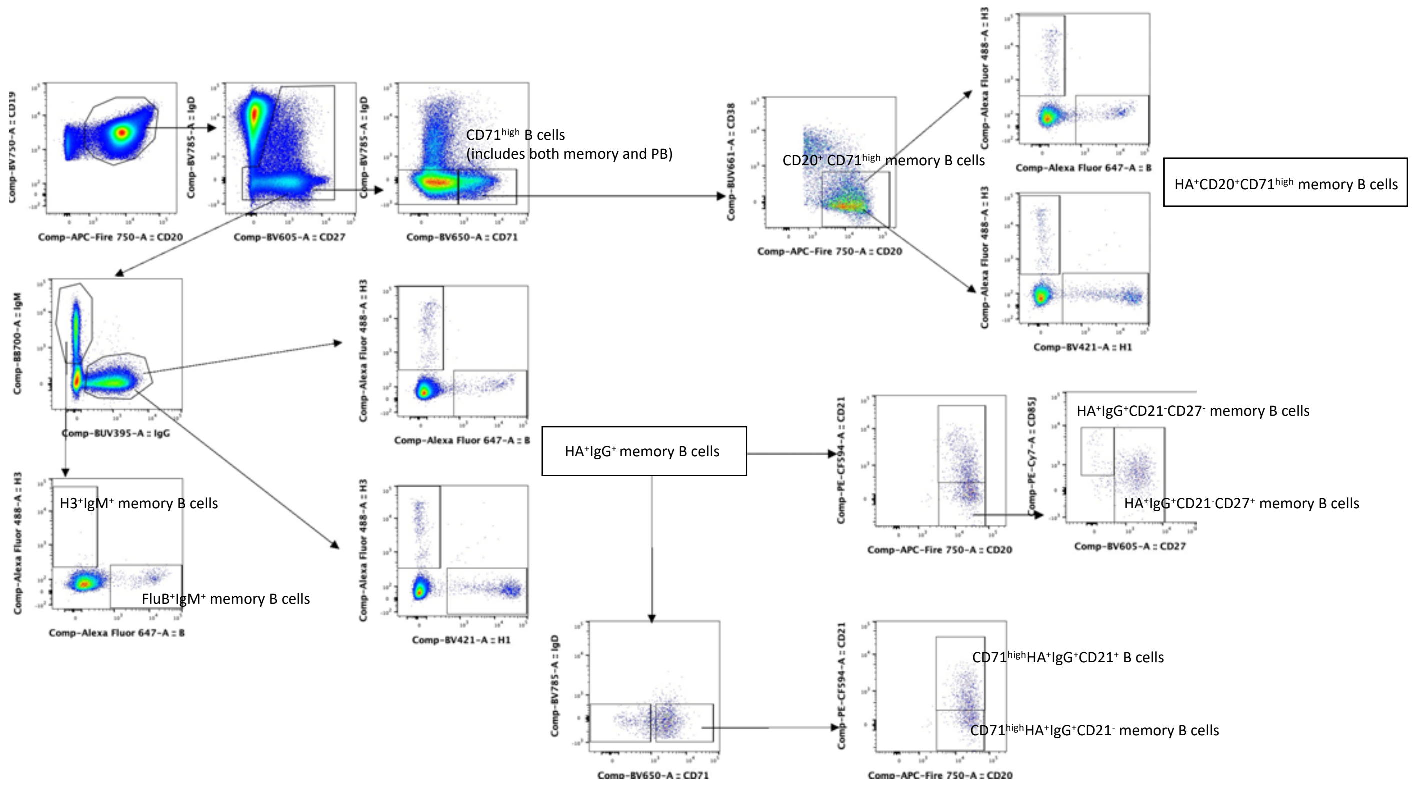

medRxiv preprint doi: https://doi.org/10.1101/2022.02.17.22271138; this version posted February 22, 2022. The copyright holder for this preprint (which was not certified by peer review) is the author/funder, who has granted medRxiv a license to display the preprint in perpetuity.
This article is a US Government work. It is not subject to copyright under 17 USC 105 and is also made available for use under a CC0 license.

Supplementary Information Figure 2: Gating strategies for the influenza-specific (**a**) plasmablast, and (**b**) memory B-cell populations.

medRxiv preprint doi: https://doi.org/10.1101/2022.02.17.22271138; this version posted February 22, 2022. The copyright holder for this preprint (which was not certified by peer review) is the author/funder, who has granted medRxiv a license to display the preprint in perpetuity. This article is a US Government work. It is not subject to copyright under 17 USC 105 and is also made available for use under a CC0 license.

Supplementary Information Fig. 3

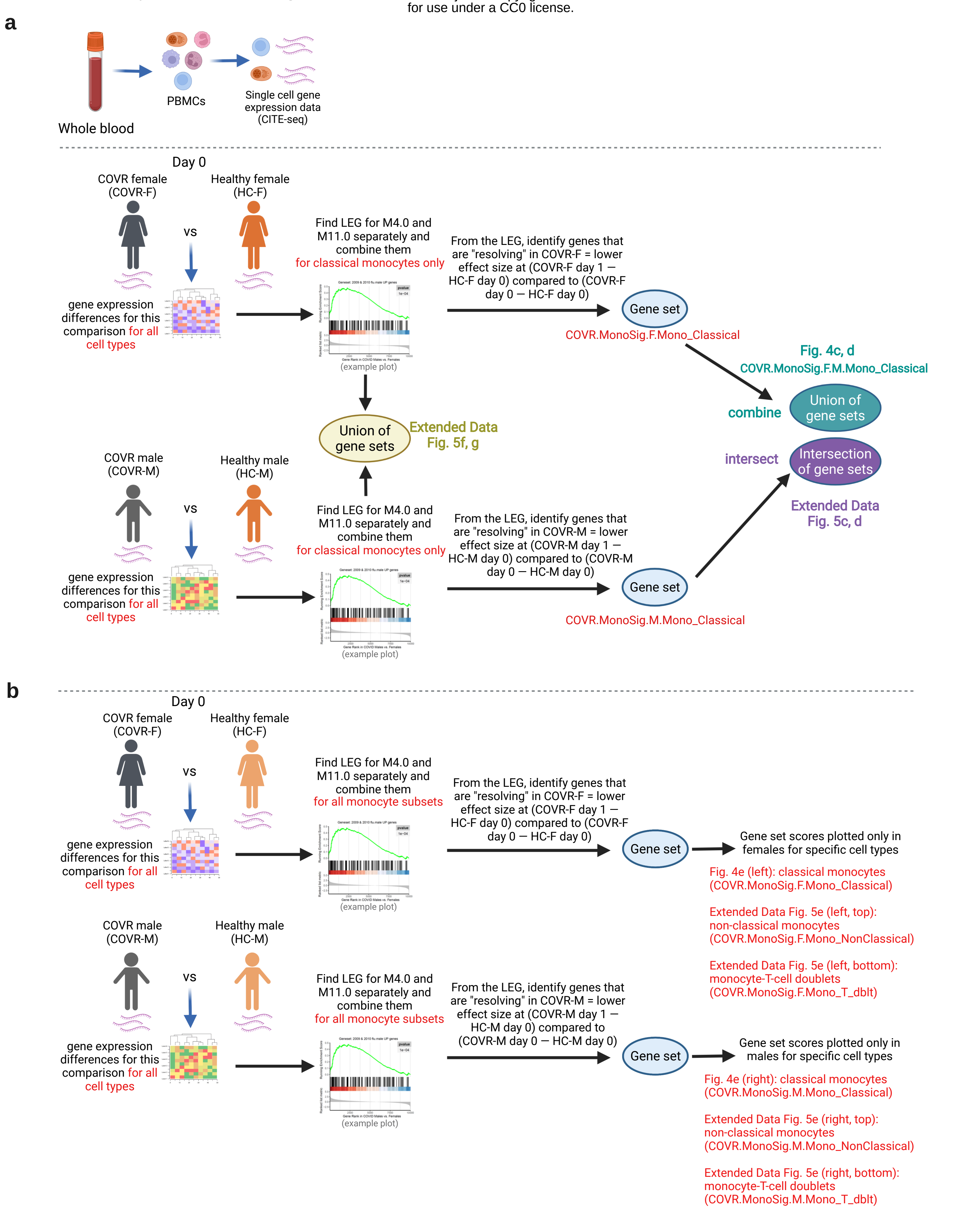

medRxiv preprint doi: https://doi.org/10.1101/2022.02.17.22271138; this version posted February 22, 2022. The copyright holder for this preprint
(which was not certified by peer review) is the author/funder, who has granted medRxiv a license to display the preprint in perpetuity.
This article is a US Government work. It is not subject to copyright under 17 USC 105 and is also made available
for use under a CC0 license.

Supplementary Information Figure 3: Schematic showing the creation of the datasets and gene sets used in the analyses shown in Fig. 4 and Extended Data Fig. 5.
