# Supplementary material for: Influenza vaccination reveals and partly reverses sex dimorphic immune imprints associated with prior mild COVID-19
Source: medRxiv. 2022 Feb 22:2022.02.17.22271138. Preprint. [Version 1] doi: 10.1101/2022.02.17.22271138 (PMC8887138; doi:10.1101/2022.02.17.22271138)
Supplement: Supplementary file 2 [file media-1.pdf]

Extended Data Table 1. Cohort Characteristics

	COVR		Healthy	
	**Female**	**Male**	**Female**	**Male**
**Subject count**	17	16	21	19
**Age (Years)**				
Median	40.2	43.7	52.5	47.6
Mean	44.9	43.7	47.2	47.4
Min	23.4	21.9	22.5	24.0
Max	70.5	67.3	70.4	69.1
**> 65 years of age**	2	2	5	5
**Race**				
Asian	1	0	2	2
Black	1	0	4	0
Multiple race	0	2	1	3
White	15	14	14	14
**Experienced vaccine side effects**	16	9	17	15
**COVID-19 Symptoms**				
Asymptomatic	1	1	-	-
Symptomatic	16	15		
**Time since COVID-19 Diagnosis (Days) ***				
Median	172.0	186.0	-	-
Mean	152.9	149.3	-	-
Min	58.0	44.0	-	-
Max	237.0	248.0	-	-
**Experienced COVID-19 residual symptoms at time of screening**	8	3	-	-
**COVID-19 Residual Symptoms**				
Brain fog	1	0	-	-
Fatigue	2	0	-	-
Loose stools	0	1	-	-
Reduced sense of taste	1	1	-	-
Reduced sense of smell / smell disturbances	5	2	-	-
Shortness of breath / Chest pressure	1	0	-	-

* Excluding asymptomatic subjects
